# Homeostatic and Hedonic Signals Differentially Regulate cFos Activation in PVN MC4R, PVN pro-TRH, PBN vGlut2, and PBN CRF Neuronal Populations

**DOI:** 10.3390/brainsci16101111

**Published:** 2026-10-15

**Authors:** Estefania Espitia-Bautista, Cinthia García-Luna, Paulina Soberanes-Chávez, Javier Berjón, Elena Álvarez-Salas, Patricia de Gortari

**Affiliations:** Laboratorio de Neurofisiología Molecular, Dirección de Investigaciones Biomédicas en Salud Mental, Instituto Nacional de Psiquiatría Ramón de la Fuente Muñiz, Calzada México-Xochimilco 101, San Lorenzo Huipulco, Tlalpan, Mexico City 14370, Mexico; estefaneb@hotmail.com (E.E.-B.); garcialuna@inprf.gob.mx (C.G.-L.); paulinasoberanes@inprf.gob.mx (P.S.-C.); javier.berjon1@gmail.com (J.B.); alvareze@inprf.gob.mx (E.Á.-S.)

**Keywords:** palatable food, stress, reward, hypothalamic paraventricular nucleus, parabrachial nucleus, limbic system

## Abstract

**Highlights:**

**What are the main findings?**

- PVNMC4R and PVNTRH neurons exhibit distinct cell-type-specific remodeling following long-term palatable diet exposure, with MC4R neurons becoming selectively tuned to hedonic refeeding under stress.
- Chronic palatable diet and stress interact to produce a “circuit sensitization” effect, wherein prior palatable exposure paradoxically enhances PBNvGlut2 and PBNCRF neuron activation during palatable refeeding in stressed animals.

**What are the implications of the main findings?**

- The PBN functions as a dynamic integrator that discriminates homeostatic from hedonic signals, but this gating function is reshaped by dietary history and stress.
- Long-term palatable food consumption flattens mesolimbic dopamine system responsiveness, uncoupling VTA dopamine and accumbal D2R expression from metabolic and stress cues, promoting persistent reward-driven feeding.

**Abstract:**

**Background**: The high worldwide prevalence of obesity, with palatable food hyperphagia as its main etiological factor, justifies studying neural circuit alterations under conditions such as chronic stress and exposure to high-energy diets, which elevate palatable food preference and body weight. Short-term intake of palatable food, acting as a satiety stimulus, activates melanocortin-4 receptor-expressing neurons in the paraventricular hypothalamic nucleus (PVN^MC4R^) and those from the caudo-lateral region of the parabrachial nucleus (PBN). It remains unclear whether stress targets this circuit, reducing satiety, or whether 2-week palatable diet exposure alters anorexigenic cells’ responsiveness to stress, such as MC4R, pro-thyrotropin-releasing hormone (pro-TRH), vesicular glutamate transporter 2 (vGlut2), and corticotropin-releasing factor (CRF)-expressing neurons. **Methods**: Male adult Wistar rats subjected to isolation stress or group-housed and fed with chow or palatable food for 2 weeks were fasted and then re-fed for 2 h with chow or chocolate milk. Using immunohistochemical analyses, we determined the circuit responsiveness to stimuli, identifying the cFos-indicated activation of anterior and medial PVN-MC4R, PVN-pro-TRH, PBN-vGlut2, and PBN-CRF-expressing cells. We also analyzed VTA tyrosine hydroxylase and accumbal type-2 dopamine receptor expression, where PBN neurons’ activity impacts and modulates the food-rewarding properties of DAergic circuit responses. **Results**: PVN^MC4R+cFos^ cells responded differently depending on food type, diet duration, combination, and nature of stimuli (homeostatic/hedonic); their responsiveness pattern was similar to PBN^vGlut2+cFos^ and different from anterior PVN^pro-TRH+cFos^ cells. **Conclusions**: The results suggest that 2-week palatable diet exposure changes satiety-circuit cells’ responsiveness, showing enhanced activity when stress and palatable food are combined, which seemed associated with their higher food intake.

## 1. Introduction

Neural circuits regulating feeding and energy homeostasis are increasingly understood as dynamic networks that integrate metabolic, visceral, sensory, emotional, and reward-related information. Rather than functioning as fixed “feeding” or “satiety” centers, hypothalamic and brainstem circuits interact with mesolimbic systems to adjust food intake according to the animal’s physiological state and environmental context [1,2,3]. Notably, prolonged exposure to obesogenic diets and environmental challenges as chronic stress can alter the responsiveness and functional organization of neural systems, controlling energy balance and food reward. These adaptations may occur at the level of neuronal excitability, synaptic transmission, and the molecular properties of specific neuronal populations [1,4].

Within this framework, the hypothalamic paraventricular nucleus (PVN)–parabrachial nucleus (PBN) pathway has emerged as an important circuit linking hypothalamic regulation of energy balance with brainstem mechanisms controlling food intake [5,6]. A relevant population comprises PVN neurons expressing melanocortin-4 receptors (MC4R), which receive input from pro-opiomelanocortin (POMC)-expressing neurons in the arcuate nucleus (ARC), a major source of α-melanocyte-stimulating hormone (α-MSH) who is involved in melanocortin-mediated control of feeding [7,8]. PVN^MC4R^ neurons also project to the lateral-caudal parabrachial nucleus (cLPBN) [6], providing a pathway through which hypothalamic metabolic information can influence brainstem circuits involved in the termination of palatable food intake [9]. The PBN is also functionally connected with the forebrain regions involved in motivational and reward-related processes, including the ventral tegmental area (VTA) [10,11,12].

Acute exposure to palatable food increases energy reservoirs, activates PVN^MC4R^-associated PBN neurons and promotes satiety, whereas inhibition of the PBN increases palatable food intake in rodents [9]. These findings are further evidence indicating that PVN^MC4R^–PBN signaling participates in physiological feeding suppression [5,6,13]. However, effects of chronic exposure to highly palatable food on this circuit have not been described.

Moreover, stress or threat conditions activate PBN-to-VTA signaling, thereby conveying negative effects that suppress food-seeking behavior, thus providing a link between emotional state and the motivational control of feeding [12]. Therefore, the outcome on feeding behavior seems to depend on the response of this pathway to the animal’s metabolic state, but also to negative stimuli exposure as well as to previous experiences. Feeding circuits integrate current energy availability with the sensory and motivational properties of food, and prolonged dietary exposure can modify their responsiveness [1,2]. Thus, the same food-related stimulus may recruit feeding circuits differently depending on whether animals are hungry or satiated as well as on their previous exposure to palatable food.

The integrative function of this network is particularly relevant because homeostatic and hedonic regulation of feeding are closely interconnected. PVN neurons receive information from ARC POMC neurons and melanocortin signaling [7], while fasting decreases α-MSH synthesis and release into the PVN, which in turn prevents anorexigenic MC4R function from activating, leading animals to eat. Stressful conditions can either suppress or promote feeding depending on the nature, intensity, duration, and chronicity of the stressor, as well as on the metabolic context in which stress occurs [14,15,16].

At the same time, hypothalamic and brainstem circuits interact with mesolimbic dopamine systems involved in the motivational and reinforcing properties of food. Chronic exposure to highly palatable food has been associated with adaptations in dopamine signaling and striatal dopamine receptors regulation, although these changes may also vary according to metabolic state [1,17]. Therefore, changes in downstream dopaminergic markers may accompany adaptations in feeding circuits without necessarily indicating a simple reduction in reward function.

PBN participates in distinct forms of feeding suppression. In addition to physiological satiety, PBN neurons expressing calcitonin gene-related peptide (CGRP) mediate anorexia associated with gastrointestinal malaise and other pathological states [18,19]. Acute stress is known to reduce feeding by activating PBN–VTA neurons [12]. In contrast, PBN neurons receiving input from PVN^MC4R^ neurons participate in physiological satiety due to homeostatic regulatory processes; they are located in the cLPBN, where they differ from the CGRP-expressing population associated with sickness responses [9] and from those activated by acute stress [12]. This functional organization suggests that PBN neurons can discriminate among different signals that suppress feeding.

Chronic stress may be particularly relevant because glucocorticoid signaling can influence food preference, energy balance, and motivated behavior. Consequently, stress may alter the responsiveness of feeding circuits to subsequent metabolic and food-related stimuli by acting on different brain regions (PVN, PBN, VTA). It remains unknown whether chronic exposure to palatable food or stress modifies the context-dependent cFos-defined activation. It also remains unclear whether fasting and stress, both of which can modify hypothalamic feeding-related activity, can alter the cFos-defined recruitment of PVN^MC4R^-associated PBN neurons during refeeding. Thus, we used a 48-h fasting period to generate a standardized state of acute energy deficit and strong feeding drive, allowing us to determine whether chronic stress alters the ability of the circuit to respond to a subsequent metabolic challenge.

We hypothesized that although fasting would increase food intake during refeeding, chronic stress might modify cFos activation of PVN^MC4R^–PBN neurons in response to this increased metabolic demand. Furthermore, because palatable food provides additional hedonic signals during refeeding, we expected that the neuronal response to fasting followed by palatable food refeeding would differ from that elicited by chow, particularly after prolonged exposure to palatable food and/or chronic stress. Likewise, it is unknown whether prolonged palatable food consumption, alone or combined with chronic stress, changes the response of this pathway to palatable versus non-palatable food and whether such changes are accompanied by adaptations in downstream mesolimbic dopamine-related markers.

Therefore, we aimed to investigate the effects of prolonged palatable food exposure and chronic isolation stress on feeding behavior and the cFos-defined recruitment of neural populations associated with the PVN^MC4R^–PBN pathway and downstream mesolimbic dopamine signaling. Adult male Wistar rats were maintained for 2 weeks under control housing or chronic isolation stress and were provided either standard chow or a palatable diet consisting of chocolate milk plus chow. Daily energy intake was recorded throughout the exposure period. Following this 2-week exposure, animals underwent a 48-h fasting period and were subsequently re-fed for 2 h at the beginning of their activity phase with either chow or palatable food. This design allowed us to examine neuronal responses under different combinations of dietary history, stress exposure, energy deficit, and refeeding stimulus.

We hypothesized that prolonged palatable food exposure and chronic stress would alter the responsiveness of PVN^MC4R^-associated feeding circuits to metabolic and food-related stimuli. Specifically, we examined whether cFos expression on PVN^MC4R^ and PBN neurons varied according to the type of refeeding stimulus and to the animals’ previous dietary and stress experience, and whether these responses were accompanied by changes in markers of VTA and NAc dopaminergic signaling. By examining these responses across different metabolic and environmental conditions, we sought to determine whether chronic palatable food consumption and stress are associated with context-dependent adaptations in neural populations involved in the integration of satiety and feeding motivation.

## 2. Materials and Methods

### 2.1. Animals

Two different sets of male Wistar rats (n = 160, 250 ± 10 g of b.w.) were obtained from the Instituto Nacional de Psiquiatría Ramón de la Fuente Muñiz (INPRFM) *vivarium*. The first set consisted of n = 96 rats and the second one of n = 64, as they were sacrificed by transcardial perfusion or by decapitation, respectively. All procedures followed the Mexican Official Norm (NOM-062-ZOO-1999) and were approved by the Institutional Ethics Committee (CEI/C/039/2023). Rats were group-housed under controlled conditions (24 ± 1 °C, inverted 12/12 h light/dark cycle, lights on at 19:00 h) for 1 week of acclimation. Water and standard chow (5001, PMI Nutrition International, Brentwood, CA, USA) were provided ad libitum.

After acclimation, each set of rats was randomly assigned (using Excel) into two groups with different housing conditions: control rats (C), which were pair-housed (n = 48 or 32/group), and stress rats (S), which were singly housed (n = 48 or 32/group). The chronic stress model used was isolation housing; this model induces an increase in serum corticosterone concentration and causes palatable food hyperphagia and increased body weight [20,21]. These groups were subdivided into 4 subgroups that were fed either chow (Standard chow 5001, 3.35 kcal/g, 28.8% protein, 13.4% fat, 56.7% carbohydrates) or palatable food (chocolate milk prepared with lactose-free milk (Alpura, Ecatepec, Mexico, 0.72 kcal/g, 16.1% protein, 37.6% fat, 46% carbohydrates) plus chow pellets. Thus, four groups per rat set (Figure 1A) were maintained under the following conditions for 2 weeks:

1. Control-chow (C-c): Pair-housed rats fed with standard chow (n = 40; 24 for immunohistochemistry and 16 for Western blot). For food intake analysis only, the cage (2 animals) was the unit, thus n = 12.
2. Control-chocolate milk (C-pal): Pair-housed rats fed with chocolate milk plus standard chow pellets (n = 40; 24 for immunohistochemistry and 16 for Western blot). For food intake analysis only, the cage (2 animals) was the unit, thus n = 12.
3. Stress-chow (S-c): Single-housed rats fed with standard chow (n = 40; 24 for immunohistochemistry and 16 for Western blot). For food intake analysis only, the cage (2 animals) was the unit, thus n = 12.
4. Stress-chocolate milk (S-pal): Single-housed rats fed with chocolate milk plus standard chow pellets (n = 40; 24 for immunohistochemistry and 16 for Western blot. For food intake analysis only, the cage (2 animals) was the unit, thus n = 12.

Food intake and body weight (b.w.) were recorded daily at 9:00 h. Chow pellets were weighed before being offered to the rats; chocolate milk was prepared twice a day and freshly offered at the beginning of the light and dark phases. Cumulative daily food intake was quantified at the times of the beverage (chocolate milk) changes at 7:00 h and 19:00 h. All groups had ad libitum access to water. For pair-housed rats, daily food intake was recorded over the 2 weeks of the experiment across the four groups as described above. As the control animals were housed in pairs, the food intake of each cage was considered as a single experimental unit (n = 12/group); in contrast, the daily food consumption of every two individually housed (stressed) rats was randomly paired and summed for comparison against controls. Here, we report the b.w. and food intake of the set of animals that were euthanized by transcardial perfusion whose brains were used for immunohistochemical analyses, since data from the decapitated rats have been published previously [22].

After 2 weeks, each group (C-c, C-pal, S-c, S-pal) from each set of rats was subdivided into four subgroups (n = 10/subgroup) depending on feeding conditions (Figure 1B): ad libitum (Al), fasting (F), fasting and chow refeeding (Rc), fasting and pal-food refeeding (Rpal). In order to euthanize all the animals on the same day and at approximately the same time, the ad libitum (Al) subgroup was kept in the same conditions for an additional 50 h following the 2-week period. This accounted for the 48 h fasting period plus the 2 h refeeding period in the fasted (F) subgroups, bringing the total experimental time period to 2 weeks and 50 h. The F subgroups were fasted for 48 h and euthanized immediately after. The rest of the F subgroups were re-fed chow or palatable food for 2 h, forming the Rc subgroup when re-fed with chow, or the Rpal subgroup when re-fed with palatable food and euthanized immediately after. A set of animals (total n = 64) was euthanized by decapitation, and their brains were used for protein expression determination using Western blot (resulting in n = 4/group). The remaining rats (total n = 96) were transcardially perfused, and their tissues were used to perform immunohistochemistry (resulting in n = 6/group) (Figure 1C,D). The sample sizes of n = 6 for immunohistochemistry and n = 4 for Western blot provide a statistical power of >99% and >93%, respectively (at α = 0.05), to detect the large biological effect sizes (d ≥ 2.95) typically observed in the food-induced brain activation literature [9,22].

Before euthanasia, food consumption was recorded for 2 h under ad libitum or refeeding conditions (chow or palatable diet). Energy intake was calculated using the amount eaten/cage (C or S; n = 3).

The subgroups formed were as follows:

1. Control-chow-fed rats (2w-chow-C; see Figure 1B,D)
  1.1. 2w-chow-C-Al group: Pair-housed animals (non-stressed, C) fed with standard chow (C) and fed ad libitum (Al). (n = 10; 6 for immunohistochemistry, 4 for Western blot).
  1.2. 2w-chow-C-F group: Pair-housed animals (non-stressed) fed with standard chow, fasted (F) for 48 h and euthanized (n = 10; 6 for immunohistochemistry, 4 for Western blot); see Figure 1.
  1.3. 2w-chow-C-Rc group: Pair-housed animals (non-stressed) fed with standard chow. These animals were fasted (F) for 48 h, re-fed with regular chow (Rc) for 2 h, and then euthanized (n = 10; 6 for immunohistochemistry, 4 for Western blot).
  1.4. 2w-chow-C-Rpal: Pair-housed animals (non-stressed) fed with standard chow. These animals were fasted for 48 h (F), re-fed with palatable food (chocolate milk, Rpal) for 2 h, and euthanized (n = 10; 6 for immunohistochemistry, 4 for Western blot).
2. Control-chocolate milk-fed rats (2w-pal-C; see Figure 1B,D)
  2.1. 2w-pal-C-Al group: Pair-housed rats (non-stressed) ad libitum fed with palatable food and fed ad libitum (Al). Then euthanized after 2 weeks and 50 h (n = 10; 6 for immunohistochemistry, 4 for Western blot).
  2.2. 2w-pal-C-F group: Pair-housed animals (non-stressed) fed with palatable food, fasted (F) for 48 h and euthanized (n = 10; 6 for immunohistochemistry, 4 for Western blot).
  2.3. 2w-pal-C-Rc group: Pair-housed animals (non-stressed) fed with palatable food. These animals were fasted for 48 h, re-fed with regular chow for 2 h, and then euthanized (n = 10; 6 for immunohistochemistry, 4 for Western blot).
  2.4. 2w-pal-C-Rpal group: Pair-housed animals (non-stressed) fed with palatable food. These animals were fasted for 48 h, re-fed with chocolate milk for 2 h, and then euthanized (n = 10; 6 for immunohistochemistry, 4 for Western blot).
3. Stress-chow-fed rats (2w-chow-S; see Figure 1B,D)
  3.1. 2w-chow-S-Al group: Single-housed animals (stressed) ad libitum fed with standard chow and non-fasted (n = 10; 6 for immunohistochemistry, 4 for Western blot).
  3.2. 2w-chow-S-F group: Single-housed animals (stressed) fed with standard chow and fasted for 48 h (n = 10; 6 for immunohistochemistry, 4 for Western blot).
  3.3. 2w-chow-S-Rc group: Single-housed animals (stressed) fed with regular chow. These animals were fasted for 48 h, re-fed with standard chow for 2 h, and euthanized (n = 10; 6 for immunohistochemistry, 4 for Western blot).
  3.4. 2w-chow-S-Rpal group: Single-housed animals (stressed) fed with standard chow. These animals were fasted for 48 h, re-fed with palatable food for 2 h, and euthanized (n = 10; 6 for immunohistochemistry, 4 for Western blot).
4. Stress-chocolate milk-fed rats (2w-pal-S; see Figure 1B,D)
  4.1. 2w-pal-S-Al group: Single-housed animals (stressed) ad libitum fed with palatable food (n = 10; 6 for immunohistochemistry, 4 for Western blot).
  4.2. 2w-pal-S-F group: Single-housed animals (stressed) fed with palatable food and euthanized after 48 h of fasting (n = 10; 6 for immunohistochemistry, 4 for Western blot).
  4.3. 2w-pal-S-Rc group: Single-housed animals (stressed) fed with palatable food. These rats were fasted for 48 h, re-fed with standard chow for 2 h, and euthanized (n = 10; 6 for immunohistochemistry, 4 for Western blot).
  4.4. 2w-pal-S-Rpal group: Single-housed animals (stressed) fed with palatable food. These rats were fasted for 48 h, re-fed with palatable food for 2 h, and euthanized (n = 10; 6 for immunohistochemistry, 4 for Western blot).

### 2.2. Blinding

Blinding was not possible during animal handling and euthanasia due to visible differences in housing (single vs. pair-housed) and diet. However, tissue analyses were conducted using coded samples to minimize researcher bias. Additionally, researchers conducting Western blot analysis and immunohistochemical cell counting were blinded to the treatment conditions, and IHC analysis was performed independently by two researchers, and their results were averaged for the final analysis.

### 2.3. Tissue Preparation

At the designated time for euthanasia, one set of rats was decapitated, and their brains were rapidly excised, rinsed with 0.9% saline solution, and frozen on dry ice. Thereafter, the brains were manually dissected, and the VTA (−4.68–−5.04 mm from bregma) and NAc (1.32–2.32 mm from bregma) were obtained and stored at −70 °C until Western blot was performed [23]. The other set of animals was deeply anesthetized with isoflurane (Fluriso, VetOne, Boise, ID, USA) and transcardially perfused with 0.9% saline, followed by 4% paraformaldehyde (PFA) in 0.1 mM phosphate buffer (pH 7.2). Brains were excised, post-fixed in 4% PFA with 10% sucrose for 24 h and cryoprotected in phosphate-buffered saline (PBS) with 20% sucrose for 24 h, followed by another 24 h in PBS with 20% sucrose at 4 °C. Afterwards, brains were frozen on dry ice and stored at −70 °C until further processing. Four series of frozen coronal brain sections (30 μm) were cut throughout the rostrocaudal extent of the hypothalamic paraventricular nucleus (PVN) [−1.08 to −2.16 mm from bregma] and caudolateral parabrachial nucleus (cLPBN) [−8.76 to −9.36 mm from bregma] using a sliding microtome. Sections were stored in a cryoprotectant solution (50% 0.05 M sodium phosphate buffer, 30% ethylene glycol, 20% glycerol) until immunohistochemistry was performed.

### 2.4. Immunohistochemistry (IHC)

For immunohistochemical analysis, brain sections were removed from the cryoprotectant solution, washed with Tris-buffered saline (TBS, pH 7.4) five times for 5 min each at room temperature (RT). Then, sections were incubated in a citrate buffer (10 mM sodium citrate, 0.05% *v*/*v* Tween-20, pH 6.0) at 65 °C for 5 min for antigen retrieval and rinsed twice with distilled water at RT. Afterwards, sections were incubated in a peroxidase solution (10% *v*/*v* H_2_O_2_ in TBS) for 10 min at RT for autofluorescence quenching, washed with TBS for 5 min, and blocked with a TBS-blocking solution containing 5% *v*/*v* normal goat serum (NGS, Cat. 005-000-121, Jackson ImmunoResearch, West Grove, PA, USA) and 0.5% *v*/*v* Triton-X 100 (Sigma-Aldrich, St. Louis, MO, USA) in TBS for 30 min. Finally, sections were processed for double or triple fluorescent IHC.

Double IHC: One series of PVN sections (3 sections per rat) was incubated in a solution containing a mouse monoclonal anti-cFos primary antibody (1:2000, Cat. ab208942, Abcam, Cambridge, UK) and a rabbit polyclonal anti-MC4R primary antibody (1:1200, Cat. PA5-20759, Invitrogen, Carlsbad, CA, USA) diluted in TBS-blocking solution. A second PVN series (3 sections per animal) was incubated in TBS-blocking solution containing a mouse monoclonal anti-cFos primary antibody (1:2000, Abcam) and a rabbit polyclonal anti-pro-TRH primary antibody (1:1200, Cat. ABN1658, Millipore, Burlington, MA, USA) for 72 h at 4 °C. The sections were then washed 5 times in TBS for 5 min each and incubated for 2 h at RT in a cocktail of goat anti-rabbit Alexa Fluor 488 secondary antibody (1:1000, Cat. 111-545-003, Jackson ImmunoResearch) and donkey anti-mouse Alexa Fluor 594 secondary antibody (1:1000, Cat. 715-585-150, Jackson ImmunoResearch) diluted in TBS-blocking solution.

Triple IHC: One series from the PBN was incubated for 72 h at 4 °C in a cocktail containing a mouse monoclonal anti-cFos primary antibody (1:2000, Abcam), a goat polyclonal anti-CGRP primary antibody (1:1000, Cat. PA1-85250, Invitrogen), and a rabbit polyclonal anti-vGlut2 primary antibody (1:600, Cat. A15177, Abclonal, Woburn, MA, USA) diluted in TBS-blocking solution. The second series was incubated for 72 h at 4 °C with TBS-blocking solution containing a mouse monoclonal anti-cFos primary antibody (1:2000, Abcam), a goat polyclonal anti-CGRP primary antibody (1:1000, Invitrogen), and a rabbit polyclonal anti-CRF primary antibody (1:1000, kindly donated by Dr. Paul Sawchenko). The sections were washed 5 times in TBS 5 min each and incubated for 2 h at RT in a cocktail of goat anti-rabbit Alexa Fluor 488 secondary antibody (1:1000, Jackson Immunoresearch), donkey anti-mouse Alexa Fluor 594 secondary antibody (1:1000, Jackson ImmunoResearch), and donkey anti-goat Alexa Fluor 647 secondary antibody (1:1000, Cat. 705-605-003 Jackson ImmunoResearch) diluted in TBS-blocking solution.

For double and triple IHC, once the secondary antibodies’ incubation time had elapsed, sections were washed 5 times in TBS for 5 min each, mounted onto Superfrost Plus slides, and air-dried in the dark. Once the sections were dry, all slides were coverslipped with a solution of 50% glycerol and 50% PBS and stored in the dark at −20 °C until imaging.

### 2.5. Image Acquisition and Analysis

High-power confocal Z-stack tile images were captured using an LSM900 Zeiss confocal microscope controlled by Zeiss Zen software 3.14 (Carl Zeiss Microscopy, Jena, Germany) using a 20× objective. Images were taken using a bidirectional line scan at 1024 × 1024 pixels captured at an eight-bit grayscale resolution. Each pixel was scanned twice, and the result was averaged to reduce optical noise. Appropriate emission filters were applied to prevent bleed-through between optical channels. Tile images were stitched with Zen software using a 10% overlap between frames. To construct 2D maximum intensity projections, confocal Z-stack images were captured by taking images with a frequency of 5 µm throughout the entire depth of the tissue section (30 µm) and then using the orthogonal projection tool to flatten the image. ImageJ software v1.54 [24] was used to create a composite image, including two or three fluorescent channels.

Photomicrographs were analyzed using ImageJ software; cFos, pro-TRH, MC4R, CGRP, CRF, and vGlut2-positive cells, as well as double-labeled (cFos + pro-TRH, cFos + MC4R, cFos + CRF, cFos + vGlut2) and triple-labeled (cFos + CGRP + CRF, cFos + CGRP + vGlut2) cells, were manually counted using the multi-point tool. cFos-positive nuclei were quantified by identifying 20–300 µm-sized particles. Colocalization of cFos with pro-TRH, MC4R, CRF, and vGlut2 was confirmed when nuclear signal was contained within the cytoplasm of cell bodies in z-stacks. Discrepancies exceeding 5% were resolved through re-examination of images by both observers. We analyzed 3 sections per region (PVN and cLPBN) per rat, and the average of the cell numbers was used as a single value for an experimental animal. Sections were counted by two different researchers to avoid bias during the analysis.

### 2.6. Immunoblotting

The VTA and left NAc were used to determine tyrosine hydroxylase (TH) and dopamine receptor 2 (D2R) protein expression. Each VTA and NAc was sonicated with 100 µL of RIPA lysis buffer (Abcam) with protease inhibitor (1:100, ThermoFisher Scientific, Waltham, MA, USA) to extract proteins. Tissue lysates were incubated for 20 min on ice and then centrifuged at 13,800 rpm at 4 °C for 10 min. Supernatants were collected, and the same volume of 2× Laemmli buffer (Sigma-Aldrich) was added before being denatured at 95 °C for 5 min. We used a 1 µL aliquot for protein determination by the micro-Lowry method.

Protein samples (30 µg) were loaded in 10% SDS-PAGE gels for electrophoresis and, after 1 h, transferred to Hybond-C extra nitrocellulose membranes (Cytiva Amersham, Marlborough, MA, USA). Membranes were then incubated in a blocking solution of 5% BSA in 1× PBS/Tween-20 0.05% for 1 h, and for 72 h, membranes containing VTA proteins were incubated with the primary polyclonal antibody for TH (1:1000, rabbit anti-TH, T2928, Sigma-Aldrich, St. Louis, MO, USA), and membranes containing NAc proteins were incubated with rabbit polyclonal anti-D2R antibody (1:1000, PA5-115142, Thermo Fisher Scientific) in the blocking solution. After washing with PBS/0.1% Tween-20, membranes were incubated for 1 h with the secondary antibody, goat anti-rabbit HRP conjugate (1:10,000, 6721 Abcam, Waltham, MA, USA), in 3% BSA. Membranes were then incubated with the polyclonal goat anti-actin antibody (1:1000, sc-1616, Santa Cruz Biotechnology, Inc., Dallas, TX, USA) as a loading control. Protein bands were revealed using luminol and visualized with a densitometer iBright CL1000 imaging system (Invitrogen, CA, USA). ImageJ software was used for the analysis and quantification of luminescent signals that represent the intensity of TH/actin signals expressed in arbitrary units.

### 2.7. Determination of Serum Corticosterone Content

We used 100 µL of rats’ serum to determine CORT levels with a competitive ELISA kit (Enzo Life Sciences Inc., New York, NY, USA), following the manufacturer’s instructions (sensitivity: 27 pg/mL, 8.4% and 8.2% of inter- and intra-assay variation, respectively).

### 2.8. Statistical Analysis

Data are expressed as the mean ± standard error of the mean (SEM). Body weight gain, energy intake from standard chow, energy intake from palatable food, and total energy intake along the 2-week experiment were analyzed by two-way repeated measures ANOVA (stress × type of food eaten for 2 weeks), while total energy intake during 2 h refeeding and corticosterone were analyzed by three-way repeated measures ANOVA (stress × type of food eaten for 2 weeks × fasting/type of food at refeeding). The food intake of each cage was considered as one unit because control animals were housed in pairs; in contrast, daily food consumption of every 2 individually housed (stressed) rats was randomly summed to perform appropriate comparisons vs. controls. Single, double, and triple immunopositive neurons were analyzed by two-way (control/stress × Ad libitum/fasting; control/stress × chow or palatable food refeeding) and three-way ANOVA (control/stress × Ad libitum/fasting or chow or palatable food refeeding × 2w-chow/2w-pal); when *p* < 0.05, a Tukey’s post-hoc test was performed. Data distribution was evaluated with the Shapiro–Wilk test and the assumption of homogeneity of variances was assessed using Levene’s test. When both assumptions were met, a parametric three-way ANOVA (Type II) was performed as the primary analysis, followed by Tukey’s post-hoc test when *p* < 0.05. When both assumptions were violated, an aligned rank transform (ART) ANOVA was additionally performed as a non-parametric alternative that does not require normality or homoscedasticity and allows the evaluation of main effects and interactions in factorial designs [25]. Effect sizes for ART were estimated as partial η^2^ derived from ART sums of squares and reported as point estimates, following standard practice in the ART literature [25]. For models in which only one of the two assumptions was violated, the parametric ANOVA was retained, given that the experimental groups were balanced (n = 4–6 per group) and the ANOVA is considered robust to moderate violations of homoscedasticity [26,27]. For repeated-measures ANOVAs, the assumption of sphericity was assessed using Mauchly’s test and was met (*p* > 0.05); therefore, no corrections to the degrees of freedom were required. Regarding the possibility of a type 1 error due to inflation from multiple comparisons, we must note that each neuronal population and brain region constitutes an independent statistical set of data; i.e., no multiple comparisons were made on each of them. The analyses were performed with RStudio software version 2026.05.1+225 (R Foundation for Statistical Computing, Vienna, Austria), and the following packages: car v3.1-5 for Type II ANOVA and Levene’s test; emmeans v2.0.4 for estimated marginal means and Tukey-adjusted pairwise comparisons; ARTool v0.11.2 for aligned rank transform ANOVAs; ggplot2 v4.0.2, ggsignif v0.6.4, ggpattern v1.3.1, and scales v1.4.0 for data visualization; and dplyr v1.2.1 for data manipulation.

## 3. Results

### 3.1. Weight Gain During the First 2 Weeks of the Experiment

Over the 2-week protocol, the 2w-chow-C groups gained significantly less weight than the 2w-pal-C and 2w-pal-S groups, as reflected by their lower growth slopes (2w-chow-C: 2.64 ± 0.08; 2w-chow-S: 3.28 ± 0.07; 2w-pal-C: 5.18 ± 0.16; 2w-pal-S: 4.85 ± 0.13). Furthermore, we could observe that both stressed groups (2w-chow-S and 2w-pal-S) gained more weight than their respective controls. The three-way repeated-measures ANOVA showed a significant interaction between diet x stress x time, F_(14,1288)_ = 2.74, *p* = 0.0003 (Figure 2A).

### 3.2. Body Weight at the End of 2 Weeks

At the end of the protocol, rats with access to palatable food gained more weight than those with only chow (2w-pal-C: 97.7% and 2w-pal-S: 88.2% vs. that of the 2w-chow-C group’s weight gain, respectively), regardless of their stress conditions. The two-way ANOVA showed a significant effect of 2w-diet, F_(1,92)_ = 27.68, *p* < 0.001 (Figure 2B).

### 3.3. Energy Ingested over 2 Weeks

The group with 2w-pal-C food had a higher kcal intake (35.7% more) over the 2 weeks, than the 2w-chow-C group. The 2w-pal-S group ingested 17% more kcal than the 2w-pal-C group, indicating a significant effect of stress on palatable food intake. The two-way repeated-measures ANOVA showed a significant effect of stress (F_(1,44)_ = 15.87, *p* = 0.0003) and diet (F_(1,44)_ = 180.9, *p* < 0.0001), and a significant interaction between 2w-diet and time (F_(14,616)_ = 2.98, *p* = 0.0002), and diet and stress (F_(1,44)_ = 8.17, *p* = 0.006) (Figure 2C).

### 3.4. Refeeding

After 48 h of fasting, all 2w-chow-fed rats increased their amount of kcal ingestion by an average of 210%—during the first 2 h of being re-fed with chow food (Rc) or palatable (Rp)—vs. the amount consumed before fasting during 2 h of the last day of the experiment (2w-chow-C-Al and 2w-chow-C-Al on average). All groups re-fed with pal and previously fed 2 weeks with palatable food increased their consumption by 193% (on average) in comparison with 2w-chow-C-Al. In contrast, the ones re-fed with chow (2w-pal-C-Rc and 2w-pal-S-Rc) did not present any change in their kcal intake when compared to their respective ad libitum control groups (Figure 2D). The results suggest that rats that were fed pal-food for 2 weeks were, in general, less motivated to eat chow food after fasting, independently of their stress condition, than those eating chow pellets for 2 weeks.

By comparing the feeding behavior among the four subgroups of the 2w-chow-fed animals in the 2 h refeeding period, we observed that neither stress exposure nor the type of food consumed induced different kcal intake (Figure 2D). In contrast, the 2w-pal-fed-Rc group showed lower amounts of kcal ingestion than animals re-fed with palatable food (2w-chow-S-Rp 54%, 2w-pal-C-Rp 38%, and 2w-pal-S-Rpal 42% more than 2w-pal-C-Rc). By performing a three-way ANOVA to compare changes between the 2w-chow and 2w-pal-fed groups (2w-diet, stress, and refeeding food type), we found a significant and large effect of refeeding food type (F_(2,24)_ = 19.99, *p* < 0.001; η^2^ (partial) = 0.62, 95% CI [0.39, 1.00]). Moreover, the interaction between refeeding and 2w-diet was statistically significant and large (F_(2,24)_ = 14.39, *p* < 0.001; η^2^ (partial) = 0.55, 95% CI [0.29, 1.00]).

### 3.5. Corticosterone Serum Levels

#### 3.5.1. Corticosterone Serum Levels in Ad Libitum and Fasted Animals from 2w-chow and 2w-pal Fed Groups

Figure 3A shows that in both groups, either fed with chow or with palatable food for 2 weeks, the isolation stress and fasting increased corticosterone serum levels to 142% and 242% vs. their respective controls (2w-chow-C-Al and 2w-pal-C-Al). The three-way ANOVA showed that the main effect of fasting was statistically significant and large (F_(1,40)_ = 25.74, *p* < 0.001; η^2^ (partial) = 0.39, 95% CI [0.20, 1.00]), and the main effect of stress was statistically significant and large (F_(1,40)_ = 8.77, *p* = 0.005; η^2^ (partial) = 0.18, 95% CI [0.04, 1.00]).

#### 3.5.2. Corticosterone Serum Levels in 2w-chow and 2w-pal Fed Groups After Refeeding

Figure 3B depicts that during refeeding, stress is not inducing corticosterone elevation independently of the 2w-diet that animals ate, since hormone levels were similar between C and S animals from both the 2w-chow and the 2w-pal fed groups. We also observed that refeeding with palatable food reduced corticosterone serum levels when compared to their respective chow-re-fed groups. The three-way ANOVA showed that the main effect of 2w-diet was statistically significant and large (F_(1,40)_ = 10.48, *p* = 0.002; η^2^ (partial) = 0.21, 95% CI [0.05, 1.00]); the main effect of refeeding was statistically significant and large (F_(1,40)_ = 50.39, *p* < 0.001; η^2^ (partial) = 0.56, 95% CI [0.38, 1.00]); and the interaction between 2w-diet, refeeding and stress was statistically significant and medium (F_(1,40)_ = 4.59, *p* = 0.038; η^2^ (partial) = 0.10, 95% CI [3.12 × 10^−3^, 1.00]).

### 3.6. Activation of the PVN–PBN Brain Circuit of Animals with 2-Week Palatable Diet Exposure, by Stress, Fasting and by Refeeding with Two Different Types of Food

#### 3.6.1. MC4R

##### Total Immunoreactive MC4R Cells in the aPVN

Supplementary Figure S1A depicts total immunoreactive MC4R-positive cells in the aPVN of 2w-chow and 2w-pal-fed maintained ad libitum or fasted, under control or stress exposure, showing a strong decrease in all groups (to 30% on average) in all groups with different conditions known to exacerbate appetite (stress and fasting) vs. that of the control group (2w-chow-C-Al). There was a dramatic decrease in the number of immunoreactive MC4R cells in all the 2w-pal-fed groups vs. that of the 2w-chow-C-Al to only 16% on average, surprisingly, including values of the 2w-pal-C-Al (Supplementary Figure S1A). The three-way ANOVA showed the main effect of 2w-diet was statistically significant and large (F_(1,40)_ = 178.65, *p* < 0.001; η^2^ (partial) = 0.82, 95% CI [0.73, 1.00]); the main effect of fasting was statistically significant and large (F_(1,40)_ = 59.53, *p* < 0.001; η^2^ (partial) = 0.60, 95% CI [0.43, 1.00]); the main effect of stress was statistically significant and large (F_(1,40)_ = 44.02, *p* < 0.001; η^2^ (partial) = 0.52, 95% CI [0.34, 1.00]); the interaction between 2w-diet and fasting was statistically significant and large (F_(1,40)_ = 25.03, *p* < 0.001; η^2^ (partial) = 0.38, 95% CI [0.19, 1.00]); the interaction between 2w-diet and stress was statistically significant and large (F_(1,40)_ = 48.71, *p* < 0.001; η^2^ (partial) = 0.55, 95% CI [0.37, 1.00]); the interaction between fasting and stress was statistically significant and large (F_(1 40)_ = 44.12, *p* < 0.001; η^2^ (partial) = 0.52, 95% CI [0.34, 1.00]); the interaction between 2w-diet, fasting and stress was statistically significant and large (F_(1,40)_ = 38.77, *p* < 0.001; η^2^ (partial) = 0.49, 95% CI [0.31, 1.00]).

After refeeding, the number of immunoreactive MC4R-positive cells in all groups, stressed or unstressed and re-fed with chow or pal-food, were similar to those in the 2w-chow-C-Rc fed animals. There was only a slight decrease at 69% in the 2w-chow-C-Rp group when compared to the stressed group 2w-chow-S-Rp) (Supplementary Figure S1B). The immunoreactive MC4R-positive cells of the 2w-pal-C-Rc group were 22% lower than those of the 2w-chow-C-Rc. Furthermore, the stressed group 2w-pal-S-Rc had lower numbers of MC4R-positive cells at 35% vs. its similar 2w-chow-S-Rp group (Supplementary Figure S1B). The three-way ANOVA showed the main effect of 2w-diet was statistically significant and large (F_(1,40)_ = 32.78, *p* < 0.001; η^2^ (partial) = 0.45, 95% CI [0.26, 1.00]); and the main effect of stress was statistically significant and large (F_(1,40)_ = 19.92, *p* < 0.001; η^2^ (partial) = 0.33, 95% CI [0.14, 1.00]).

##### Total Immunoreactive MC4R Cells in the mPVN

Supplementary Figure S1C shows the immunoreactive MC4R-positive cells in the mPVN of 2w-chow-fed and 2w-pal-fed rats kept ad libitum or fasted, under control or stress exposure. Values of immunoreactive MC4R-positive cells in the 2w-chow-C-Al groups were higher (92.3%) than those observed in the aPVN of the same animals (aPVN = 134, mPVN = 258), although we did not compare them using statistical analyses. Fasting, stress, and their combination, as expected, reduced the number of these cells in the mPVN of the 2w-chow-fed rats by 38% vs. Al (=100%), similarly among the three groups of the 2w-chow (Supplementary Figure S1C). When comparing 2w-pal-fed animals with the 2w-chow-C-Al group, the number of reduced immunoreactive cells decreased to 36.5%. As in the aPVN, the MC4R neurons decreased in the mPVN of the 2w-pal-C-Al vs. the 2w-chow-C-Al animals (Supplementary Figure S1C). The three-way ANOVA showed the main effect of 2w-diet was statistically significant and large (F_(1,40)_ = 63.45, *p* < 0.001; η^2^ (partial) = 0.61, 95% CI [0.45, 1.00]); the main effect of fasting was statistically significant and large (F_(1,40)_ = 10.10, *p* = 0.003; η^2^ (partial) = 0.20, 95% CI [0.05, 1.00]); the main effect of stress was statistically significant and large (F_(1,40)_ = 8.79, *p* = 0.005; η^2^ (partial) = 0.18, 95% CI [0.04, 1.00]); the interaction between 2w-diet and stress was statistically significant and large (F_(1,40)_ = 11.18, *p* = 0.002; η^2^ (partial) = 0.22, 95% CI [0.06, 1.00]); and the interaction between fasting and stress was statistically significant and medium (F_(1,40)_ = 5.83, *p* = 0.020; η^2^ (partial) = 0.13, 95% CI [0.01, 1.00]).

After refeeding either with Rc or Rp, the 2w-pal-fed animals, stressed or unstressed, showed a lower number of immunoreactive cells for MC4R than those of the 2w-chow-C-Al group before fasting and refeeding (Supplementary Figure S1D). The three-way ART-type ANOVA showed that the main effect of 2w-diet was statistically significant and large (F_(1,39)_ = 8.33, *p* = 0.00633; η^2^ (partial) = 0.18); the main effect of refeeding was statistically significant and large (F_(1,39)_ = 17.09, *p* < 0.001; η^2^ (partial) = 0.30); and the main effect of stress was statistically significant and large (F_(1,39)_ = 13.77, *p* < 0.001; η^2^ (partial) = 0.26).

##### aPVN^MC4R^-cFos Cells in 2w-chow and 2w-pal Ad Libitum and Fasted Rats

Figure 4A shows that the number of activated cells in aPVN^MC4R^ (positive for MC4R and cFos) of 2w-chow-C-Al animals was on average 10 ± 1.42 (=100%), which decreased by fasting them to 30% and by being exposed to stress to 10.5%. The reduction to ~20% was also observed in the 2w-pal-C-AL and 2w-pal-S-AL groups, and in the fasted subgroups (2w-pal-C-F to 10% and 2w-pal-S-F to ~0%) (Figure 4A and Figure 5). The three-way ANOVA showed that the main effect of 2w-diet was statistically significant and large (F_(1,40)_ = 36.50, *p* < 0.001; η^2^ (partial) = 0.48, 95% CI [0.29, 1.00]); the main effect of fasting was statistically significant and large (F_(1,40)_ = 16.67, *p* < 0.001; η^2^ (partial) = 0.29, 95% CI [0.11, 1.00]); the main effect of stress was statistically significant and large (F_(1,40)_ = 37.38, *p* < 0.001; η^2^ (partial) = 0.48, 95% CI [0.30, 1.00]); the interaction between 2w-diet and fasting was statistically significant and large (F_(1,40)_ = 10.04, *p* = 0.003; η^2^ (partial) = 0.20, 95% CI [0.05, 1.00]); the interaction between 2w-diet and stress was statistically significant and large (F_(1,40)_ = 24.11, *p* < 0.001; η^2^ (partial) = 0.38, 95% CI [0.18, 1.00]); the interaction between fasting and stress was statistically significant and large (F_(1,40)_ = 12.89, *p* < 0.001; η^2^ (partial) = 0.24, 95% CI [0.07, 1.00]); and the interaction between 2w-diet, fasting and stress was statistically significant and large (F_(1,40)_ = 14.28, *p* < 0.001; η^2^ (partial) = 0.26, 95% CI [0.09, 1.00]).

##### aPVN^MC4R^-cFos Cells in 2w-chow and 2w-pal Fed Rats After Refeeding

Figure 4B shows that after refeeding, the 2w-chow-C-Rc or Rp groups, as well as those stressed (2w-chow-S-Rc or Rp), returned to values similar to those before fasting (shown in Figure 4A) and those of the ad libitum group (2w-chow-C-AL) (around 10–15 activated cells/group). In contrast, those for 2w-pal-C-Rc and 2w-pal–Rc remained lower than those for 2w-chow-C-Rc or 2w-chow-C-Rp; the 2w-pal-C-Rc and the 2w-pal-S-Rc partially recovered the number of activated cells, and when stress and palatable food were combined, the 2w-pal-S-Rp showed a similar number of cells to the 2w-chow-C group. The three-way ART-type ANOVA showed that the main effect of 2w-diet was statistically significant and large (F_(1,40)_ = 53.85, *p* < 0.001; η^2^ (partial) = 0.57); the main effect of refeeding was statistically significant and large (F_(1,40)_ = 16.21, *p* < 0.001; η^2^ (partial) = 0.29); the main effect of stress was statistically significant and medium (F_(1,40)_ = 5.33, *p* = 0.0263; η^2^ (partial) = 0.12); the interaction between 2w-diet and refeeding was statistically significant and large (F_(1,40)_ = 15.84, *p* < 0.001; η^2^ (partial) = 0.28); and the interaction between 2w-diet, refeeding and stress was statistically significant and large (F_(1,40)_ = 13.34, *p* < 0.001; η^2^ (partial) = 0.25).

##### mPVN^MC4R^-cFos Cells in 2w-chow-Ad Libitum and Fasted Rats

Figure 4C depicts the differences between Al/fasted and S/C of the animals kept on chow or palatable food diets during 2 weeks. We observed a pattern of changes similar to those in the aPVN (Figure 4A), since fasted groups and fasted/stress groups from chow (brown and ochre tones) or palatable food diets (blue-green tones) decreased (on average) to ~14.5% of the 2w-chow-C-AL animals. Additionally, the decrease in the 2w-chow-S-Al was ~41% of 2w-chow-C-AL animals. The three-way ART-type ANOVA showed that the main effect of 2w-diet was statistically significant and large (F_(1,40)_ = 67.63, *p* < 0.001; η^2^ (partial) = 0.63); the main effect of fasting was statistically significant and large (F_(1,40)_ = 89.03, *p* < 0.001; η^2^ (partial) = 0.69); the main effect of stress was statistically significant and large (F_(1,40)_ = 52.08, *p* < 0.001; η^2^ (partial) = 0.57); the interaction between 2w-diet and fasting was statistically significant and large (F_(1,40)_ = 76.47, *p* < 0.001; η^2^ (partial) = 0.66); the interaction between 2w-diet and stress was statistically significant and large (F_(1,40)_ = 35.13, *p* < 0.001; η^2^ (partial) = 0.47); the interaction between fasting and stress was statistically significant and large (F_(1,40)_ = 43.57, *p* < 0.001; η^2^ (partial) = 0.52); and the interaction between 2w-diet, fasting and stress was statistically significant and large (F_(1,40)_ = 37.62, *p* < 0.001; η^2^ (partial) = 0.48).

##### mPVN^MC4R^-cFos Cells in 2w-chow and 2w-pal Fed Rats After Refeeding

Figure 4D also shows a similar pattern of changes among groups as in the aPVN (Figure 4B); we observed a lower number of activated cells in 2w-pal-C-Rc at 34.78% vs. all subgroups of the 2w-chow-fed animals (100% on average). We also observed an increase in the number of activated cells in 2w-chow-C-Rc, 2w-pal-C-Rc, 2w-pal-C-Rp, and 2w-pal-S-Rc when compared to rats with combined stress and fasting stimuli (2w-pal-S-F group). The three-way ANOVA showed that the main effect of 2w-diet was statistically significant and medium (F_(1,40)_ = 4.46, *p* = 0.041; η^2^ (partial) = 0.10, 95% CI [2.34 × 10^−3^, 1.00]); the main effect of refeeding was statistically significant and large (F_(1,40)_ = 7.96, *p* = 0.007; η^2^ (partial) = 0.17, 95% CI [0.03, 1.00]); the main effect of stress was statistically significant and large (F_(1,40)_ = 7.81, *p* = 0.008; η^2^ (partial) = 0.16, 95% CI [0.03, 1.00]); the interaction between 2w-diet and refeeding was statistically significant and large (F_(1,40)_ = 23.31, *p* < 0.001; η^2^ (partial) = 0.37, 95% CI [0.18, 1.00]); and the interaction between 2w-diet and stress was statistically significant and medium (F_(1,40)_ = 4.35, *p* = 0.044; η^2^ (partial) = 0.10, 95% CI [1.61 × 10^−3^, 1.00]).

#### 3.6.2. pro-TRH

##### Total TRHergic Cells in the aPVN

Supplementary Figure S2A depicts total immunoreactive pro-TRH-positive cells in the aPVN of 2w-chow and 2w-pal rats, fed ad libitum or fasted, under control or stress exposure. We observed in animals previously fed with chow that fasting and stress reduced the number of those cells (on average to 58%) vs. the control group 2w-chow-C-Al (=100%) (Supplementary Figure S2A). Interestingly, the combination of stress and fasting was unable to decrease immunoreactive pro-TRH-expressing cells, since the values were similar (105%) to those of the control group (2w-chow-C-Al) and higher (by 177%) than those of the 2w-pal-C-Al animals (Supplementary Figure S2A). The three-way ANOVA showed that the main effect of 2w-diet was statistically significant and large (F_(1,31)_ = 18.34, *p* < 0.001; η^2^ (partial) = 0.37, 95% CI [0.15, 1.00]); the main effect of fasting was statistically significant and large (F_(1,31)_ = 22.36, *p* < 0.001; η^2^ (partial) = 0.42, 95% CI [0.20, 1.00]); the main effect of stress was statistically significant and large (F_(1,31)_ = 8.10, *p* = 0.008; η^2^ (partial) = 0.21, 95% CI [0.04, 1.00]); the interaction between 2w-diet and fasting was statistically significant and large (F_(1,31)_ = 23.56, *p* < 0.001; η^2^ (partial) = 0.43, 95% CI [0.21, 1.00]); the interaction between 2w-diet and stress was statistically significant and medium (F_(1,31)_ = 4.47, *p* = 0.043; η^2^ (partial) = 0.13, 95% CI [2.46 × 10^−3^, 1.00]); the interaction between fasting and stress was statistically significant and large (F_(1,31)_ = 54.57, *p* < 0.001; η^2^ (partial) = 0.64, 95% CI [0.46, 1.00]); and the interaction between 2w-diet, fasting, and stress was statistically significant and large (F_(1,31)_ = 14.93, *p* < 0.001; η^2^ (partial) = 0.32, 95% CI [0.11, 1.00]).

When re-fed either with chow (Rc) or chocolate milk (Rp), all groups maintained a similar number of immunoreactive pro-TRH aPVN cells in both the 2w-chow and 2w-pal-fed groups. Only the 2w-pal-C-Rp rats showed lower numbers of those positive cells by 41% on average vs. the rest of the groups (100%; Supplementary Figure S2B). The three-way ANOVA showed that the main effect of 2w-diet was statistically significant and large (F_(1,30)_ = 15.49, *p* < 0.001; η^2^ (partial) = 0.34, 95% CI [0.12, 1.00]); the main effect of stress was statistically significant and large (F_(1,30)_ = 9.10, *p* = 0.005; η^2^ (partial) = 0.23, 95% CI [0.05, 1.00]); and the interaction between refeeding and stress was statistically significant and large (F_(1,30)_ = 15.43, *p* < 0.001; η^2^ (partial) = 0.34, 95% CI [0.12, 1.00]).

##### Total Immunoreactive Positive TRHergic Cells in the mPVN

Supplementary Figure S2C shows the immunoreactive pro-TRH-positive cells in the mPVN of 2w-chow- and 2w-pal-fed, kept ad libitum or fasted, under control or stress exposure. For animals previously fed with chow, fasting and stress induced the expected decrease to 54% and to 31%, respectively, when compared to the number of positive cells in the 2w-chow-C-Al group; the combined stimuli also reduced its number to 52% vs. the C-Al group (100%). In contrast, there were no decreases in the number of immunoreactive pro-TRH positive cells by fasting or stress or their combination vs. their respective control group (2w-pal-C-Al) (Supplementary Figure S2C). The three-way ANOVA suggested that: the main effect of 2w-diet was statistically significant and large (F_(1,29)_ = 34.12, *p* < 0.001; η^2^ (partial) = 0.54, 95% CI [0.32, 1.00]); the main effect of stress was statistically significant and large (F_(1,29)_ = 12.13, *p* = 0.002; η^2^ (partial) = 0.29, 95% CI [0.09, 1.00]); the interaction between 2w-diet and fasting was statistically significant and large (F_(1,29)_ = 17.32, *p* < 0.001; η^2^ (partial) = 0.37, 95% CI [0.15, 1.00]); the interaction between 2w-diet and stress was statistically significant and large (F_(1,29)_ = 15.50, *p* < 0.001; η^2^ (partial) = 0.35, 95% CI [0.13, 1.00]); the interaction between fasting and stress was statistically significant and large (F_(1,29)_ = 12.19, *p* = 0.002; η^2^ (partial) = 0.30, 95% CI [0.09, 1.00]); and the interaction between 2w-diet, fasting and stress was statistically significant and large (F_(1,29)_ = 13.05, *p* = 0.001; η^2^ (partial) = 0.31, 95% CI [0.10, 1.00]).

When re-fed only the 2w-chow-C-Rpal, the rats showed higher numbers of mPVN immunoreactive pro-TRH positive cells (182%) vs. 2w-chow-C-Rc (100%) (Supplementary Figure S2D). Values in this group (2w-chow-C-Rp) were also higher by 59% more on average than in the cell numbers of 2w-chow-S-Rc, 2w-chow-S-Rp, 2w-pal-C-Rc, and 2w-pal-C-Rp (Supplementary Figure S2D). The three-way ANOVA suggests that the main effect of refeeding was statistically significant and medium (F_(1,30)_ = 4.44, *p* = 0.044; η^2^ (partial) = 0.13, 95% CI [2.15 × 10^−3^, 1.00]); the interaction between 2w-diet and refeeding was statistically significant and large (F_(1,30)_ = 13.17, *p* = 0.001; η^2^ (partial) = 0.31, 95% CI [0.10, 1.00]); the interaction between 2w-diet and stress was statistically significant and large (F_(1,30)_ = 14.71, *p* < 0.001; η^2^ (partial) = 0.33, 95% CI [0.11, 1.00]); and the interaction between refeeding and stress was statistically significant and large (F_(1,30)_ = 9.29, *p* = 0.005; η^2^ (partial) = 0.24, 95% CI [0.05, 1.00]).

##### aPVN^pro-TRH^-cFos Cells of 2w-chow and 2w-pal Ad Libitum and Fasted Rats

Figure 6A shows the number of pro-TRH-cFos expressing cells in the aPVN of 2w-chow-fed rats and of 2w-pal-fed rats of control and stress groups maintained in ad libitum or after fasting period. We observed practically no difference in chow-fed animals by stress, fasting, or their combination; only the group of 2w-pal-S-F showed a higher number of pro-TRH-cFos cells in this subcompartment of PVN in comparison with all the other groups (Figure 6A and Figure 7). The three-way ANOVA showed that the main effect of stress was statistically significant and large (F_(1,31)_ = 9.94, *p* = 0.004; η^2^ (partial) = 0.24, 95% CI [0.06, 1.00]); the interaction between 2w-diet and fasting was statistically significant and large (F_(1,31)_ = 8.57, *p* = 0.006; η^2^ (partial) = 0.22, 95% CI [0.04, 1.00]); the interaction between fasting and stress was statistically significant and large (F_(1,31)_ = 11.92, *p* = 0.002; η^2^ (partial) = 0.28, 95% CI [0.08, 1.00]); and the interaction between 2w-diet, fasting and stress was statistically significant and large (F_(1,31)_ = 5.45, *p* = 0.026; η^2^ (partial) = 0.15, 95% CI [0.01, 1.00]).

##### aPVN^pro-TRH^-cFos Cells in 2w-chow and 2w-pal Fed Rats After Refeeding

Figure 6B depicts that after refeeding with chow (Rc) or palatable food (Rp) only in the 2w-chow-C fed animals, the number of positive cells for pro-TRH-cFos in the aPVN increased to 12–14 positive cells vs. the ad libitum control-fed rats (3 cells, Figure 6A). The stressed group re-fed with chow (2w-chow-S-Rc) had a lower number of positive cells by ~20% than 2w-chow-C-Rc and 2w-chow-C-Rp on average; also, the 2w-pal-C-Rc had a lower number of positive cells by ~35% vs. its control (2w-chow-C-Rc). Moreover, the 2w-pal-S-Rp group showed lower numbers, by 67% compared to the positive cells only vs. 2w-chow-C-Rc. The three-way ART-type ANOVA showed that the main effect of 2w-diet was statistically significant and large (F_(1,29)_ = 15.14, *p* < 0.001; η^2^ (partial) = 0.34); the main effect of stress was statistically significant and large (F_(1,29)_ = 9.78, *p* = 0.004; η^2^ (partial) = 0.25); the interaction between 2w-diet and refeeding was statistically significant and medium (F_(1,29)_ = 4.23, *p* = 0.0487; η^2^ (partial) = 0.13); the interaction between 2w-diet and stress was statistically significant and large (F_(1,29)_ = 20.48, *p* < 0.001; η^2^ (partial) = 0.41); the interaction between refeeding and stress was statistically significant and medium (F_(1,29)_ = 4.53, *p* = 0.042; η^2^ (partial) = 0.13); and the interaction between 2w-diet, refeeding and stress was statistically significant and large (F_(1,29)_ = 5.28, *p* = 0.0289; η^2^ (partial) = 0.15).

##### mPVN^pro-TRH^-cFos Cells of 2w-chow and 2w-pal Ad Libitum and Fasted Rats

Figure 6C shows that in the mPVN, the rats from the 2w-chow-C-Al group had a greater number of pro-TRH-cFos positive cells than in the aPVN (Figure 6A). Notably, the control group of the pal-fed rats (2w-pal-C-Al), in contrast, showed a lower number of positive cells in the mPVN, down to 31.6%, than the chow-control animals (2w-chow-C-Al). Figure 6 also shows that groups with fasting, stress, or their combination of the 2w-chow-fed animals, as well as those in the same conditions as the 2w-pal-fed rats, had a lower number of positive cells by ~70% on average vs. 2w-chow-C-Al rats (Figure 6C). The three-way ART-type ANOVA showed that the main effect of 2w-diet was statistically significant and large (F_(1,29)_ = 17.70, *p* < 0.001; η^2^ (partial) = 0.38); the main effect of stress was statistically significant and large (F_(1,29)_ = 68.18, *p* < 0.001; η^2^ (partial) = 0.70); the interaction between 2w-diet and fasting was statistically significant and medium (F_(1,29)_ = 4.49, *p* = 0.0428; η^2^ (partial) = 0.13); the interaction between 2w-diet and stress was statistically significant and large (F_(1,29)_ = 33.07, *p* < 0.001; η^2^ (partial) = 0.53); the interaction between fasting and stress was statistically significant and large (F_(1,29)_ = 23.95, *p* < 0.001; η^2^ (partial) = 0.45); and the interaction between 2w-diet, fasting and stress was statistically significant and large (F_(1,29)_ = 27.33, *p* < 0.001; η^2^ (partial) = 0.49).

##### mPVN^pro-TRH^-cFos Cells in 2w-chow and 2w-pal Fed Rats After Refeeding

Figure 6D shows that the groups of chow-fed animals, 2w-chow-C-Rp, 2w-chow-S-Rc, and 2w-chow-S-Rp had a similar number of pro-TRH-cFos positive cells, but only the value of 2w-chow-C-Rp was significantly higher by ~133% vs. the 2w-chow-C-Rc. All the groups of the 2w-pal-fed animals had lower numbers of positive cells vs. their respective chow-fed controls (2w-pal-C-Rp to 20%X, 2w-pal-S-Rc to 43%, and 2w-pal-S-Rp to 44%). The three-way ART-type ANOVA showed that the main effect of 2w-diet was statistically significant and large (F_(1,26)_ = 23.16, *p* < 0.001; η^2^ (partial) = 0.47); the main effect of refeeding was statistically significant and large (F_(1,26)_ = 8.08, *p* = 0.0086; η^2^ (partial) = 0.24); the main effect of stress was statistically significant and large (F_(1,26)_ = 6.94, *p* = 0.014; η^2^ (partial) = 0.21); the interaction between 2w-diet and refeeding was statistically significant and large (F_(1,26)_ = 6.01, *p* = 0.0212; η^2^ (partial) = 0.19); the interaction between refeeding and stress was statistically significant and large (F_(1,26)_ = 16.92, *p* < 0.001; η^2^ (partial) = 0.39); and the interaction between 2w-diet, refeeding and stress was statistically significant and large (F_(1,26)_ = 8.68, *p* = 0.00671; η^2^ (partial) = 0.25).

#### 3.6.3. vGlut2

##### PBN^vGlut2^-cFos (no CGRP) Cells of 2w-chow and 2w-pal Ad Libitum and Fasted Rats

Figure 8A and Figure 9 shows that the number of positive cells for vGlut2-cFos was almost negligible in all groups, except for the 2w-pal-C-F, since after fasting a mean of one cell/group was observed. The three-way ANOVA showed that the main effect of 2w-diet was statistically significant and large (F_(1,33)_ = 6.77, *p* = 0.014; η^2^ (partial) = 0.17, 95% CI [0.02, 1.00]), and the interaction between fasting and stress was statistically significant and large (F_(1,33)_ = 6.85, *p* = 0.013; η^2^ (partial) = 0.17, 95% CI [0.02, 1.00]).

##### PBN^vGlut2^-cFos (no CGRP) Cells in 2w-chow and 2w-pal Fed Rats After Refeeding

Figure 8B showed larger numbers of positive cells in various groups of the 2w-chow or pal-fed rats after refeeding when compared to fed/fasted rats (Figure 8A). The largest increase was observed in the 2w-pal-S-Rp rats to more than eight cells/group, when compared to all groups. The three-way ART-type ANOVA showed that the main effect of 2w-diet was statistically significant and large (F_(1,32)_ = 26.12, *p* < 0.001; η^2^ (partial) = 0.45); the main effect of refeeding was statistically significant and large (F_(1,32)_ = 57.52, *p* < 0.001; η^2^ (partial) = 0.64); the interaction between 2w-diet and refeeding was statistically significant and large (F_(1,32)_ = 7.94, *p* = 0.00821; η^2^ (partial) = 0.20); the interaction between 2w-diet and stress was statistically significant and large (F_(1,32)_ = 8.95, *p* = 0.0053; η^2^ (partial) = 0.22); the interaction between refeeding and stress was statistically significant and large (F_(1,32)_ = 6.89, *p* = 0.0132; η^2^ (partial) = 0.18); and the interaction between 2w-diet, refeeding and stress was statistically significant and large (F_(1,32)_ = 11.11, *p* = 0.00218; η^2^ (partial) = 0.26).

#### 3.6.4. CRF

##### PBN^CRF^-cFos (no CGRP) Cells of 2w-chow and 2w-pal Ad Libitum and Fasted Rats

Regarding Figure 8C, the three-way ANOVA showed that the interaction between 2w-diet and fasting was statistically significant and medium (F_(1,32)_ = 4.35, *p* = 0.045; η^2^ (partial) = 0.12, 95% CI [1.55 × 10^−3^, 1.00]), and the interaction between fasting and stress was statistically significant and large (F_(1,32)_ = 6.02, *p* = 0.020; η^2^ (partial) = 0.16, 95% CI [0.02, 1.00]).

##### PBN^CRF^-cFos (no CGRP) Cells in 2w-chow and 2w-pal Fed Rats After Refeeding

Figure 8D depicts that after refeeding, all the 2w-chow or pal-fed groups had detectable positive cells for CRF-cFos in the PBN; the group with the greater number of positive cells was the 2w-pal-S-Rp, showing a mean of seven cells, which was significantly higher than all the other groups. The three-way ANOVA showed that the main effect of 2w-diet was statistically significant and large (F_(1,32)_ = 20.80, *p* < 0.001; η^2^ (partial) = 0.39, 95% CI [0.18, 1.00]); the main effect of refeeding was statistically significant and large (F_(1,32)_ = 19.82, *p* < 0.001; η^2^ (partial) = 0.38, 95% CI [0.17, 1.00]); the main effect of stress was statistically significant and large (F_(1,32)_ = 7.57, *p* = 0.010; η^2^ (partial) = 0.19, 95% CI [0.03, 1.00]); the interaction between 2w-diet and refeeding was statistically significant and large (F_(1,32)_ = 21.18, *p* < 0.001; η^2^ (partial) = 0.40, 95% CI [0.18, 1.00]); the interaction between 2w-diet and stress was statistically significant and large (F_(1,32)_ = 8.79, *p* = 0.006; η^2^ (partial) = 0.22, 95% CI [0.04, 1.00]); the interaction between refeeding and stress was statistically significant and large (F_(1,32)_ = 11.82, *p* = 0.002; η^2^ (partial) = 0.27, 95% CI [0.08, 1.00]); and the interaction between 2w-diet, refeeding and stress was statistically significant and large (F_(1,32)_ = 10.55, *p* = 0.003; η^2^ (partial) = 0.25, 95% CI [0.06, 1.00]).

#### 3.6.5. Total Immunoreactive cFos Cells in the cLPBN

Supplementary Figure S3A shows that by analyzing the total number of immunoreactive cFos cells in the cLPBN, instead of only those where cFos colocalized with CRF or vGlut2, we found the same lack of effect of the homeostatic signals similarly in both 2w-chow and 2w-pal-fed groups (Supplementary Figure S3A); only the 2w-pal-C-F had an increase in comparison with 2w-pal-C-Al. The ANOVA showed that the main effect of 2w-diet was statistically significant and medium (F_(1,33)_ = 4.23, *p* = 0.048; η^2^ (partial) = 0.11, 95% CI [6.65 × 10^−4^, 1.00]); the main effect of stress was statistically significant and large (F_(1,33)_ = 5.53, *p* = 0.025; η^2^ (partial) = 0.14, 95% CI [0.01, 1.00]); and the interaction between fasting and stress was statistically significant and large (F_(1,33)_ = 6.56, *p* = 0.015; η^2^ (partial) = 0.17, 95% CI [0.02, 1.00]).

After being re-fed, the group 2w-pal-S-Rp showed a significantly higher number of immunoreactive cFos cells in the cLPBN than all the other groups (Supplementary Figure S3B). The three-way ART-type ANOVA showed that the main effect of 2w-diet was statistically significant and large (F_(1,32)_ = 30.89, *p* < 0.001; η^2^ (partial) = 0.49); the main effect of refeeding was statistically significant and large (F_(1,32)_ = 50.64, *p* < 0.001; η^2^ (partial) = 0.61); the main effect of stress was statistically significant and large (F_(1,32)_ = 7.34, *p* = 0.0108; η^2^ (partial) = 0.19); the interaction between 2w-diet and refeeding was statistically significant and large (F_(1,32)_ = 17.63, *p* < 0.001; η^2^ (partial) = 0.36); the interaction between 2w-diet and stress was statistically significant and large (F_(1,32)_ = 12.95, *p* = 0.00107; η^2^ (partial) = 0.29); the interaction between refeeding and stress was statistically significant and large (F_(1,32)_ = 12.84, *p* = 0.00111; η^2^ (partial) = 0.29); and the interaction between 2w-diet, refeeding and stress was statistically significant and large (F_(1,32)_ = 17.39, *p* < 0.001; η^2^ (partial) = 0.35).

### 3.7. Differential VTA Tyrosine Hydroxylase and NAc Dopamine Receptor Type 2 Expression in Animals with 2-Week Palatable Diet Exposure, by Stress, Fasting and by Refeeding with Two Different Types of Food

#### 3.7.1. VTA Tyrosine Hydroxylase (TH) Expression in Ad Libitum and Fasted Animals from 2w-chow and 2w-pal-fed Groups

Figure 10A and Supplementary Figure S4 shows that only the 2w-chow-S-F fed groups presented a reduced expression of TH in the VTA—of 55.74% on average—vs. all the other groups. The three-way ART-type ANOVA showed that the main effect of 2w-diet was statistically significant and large (F_(1,24)_ = 12.75, *p* = 0.00155; η^2^ (partial) = 0.35); the main effect of fasting was statistically significant and large (F_(1,24)_ = 14.15, *p* < 0.001; η^2^ (partial) = 0.37); the main effect of stress was statistically significant and large (F_(1,24)_ = 4.55, *p* = 0.0433; η^2^ (partial) = 0.16); the interaction between 2w-diet and fasting was statistically significant and large (F_(1,24)_ = 15.93, *p* < 0.001; η^2^ (partial) = 0.40); the interaction between 2w-diet and stress was statistically significant and large (F_(1,24)_ = 4.83, *p* = 0.0378; η^2^ (partial) = 0.17); and the interaction between fasting and stress was statistically significant and large (F_(1,24)_ = 15.51, *p* < 0.001; η^2^ (partial) = 0.39.

#### 3.7.2. VTA Tyrosine Hydroxylase (TH) Expression in 2w-chow and 2w-pal-fed Groups After Refeeding

Figure 10B and Supplementary Figure S4 shows that after refeeding, only the 2w-chow-C-Rp group showed a lower expression of TH in the VTA vs. the 2w-chow-S-Rc (by 49.66%) and vs. 2w-pal-C-Rp (by 52.41%). The three-way ANOVA showed that the main effect of 2w-diet was statistically significant and large (F_(1,24)_ = 4.78, *p* = 0.039; η^2^ (partial) = 0.17, 95% CI [5.14 × 10^−3^, 1.00]); the interaction between 2w-diet and refeeding was statistically significant and large (F_(1,24)_ = 9.47, *p* = 0.005; η^2^ (partial) = 0.28, 95% CI [0.06, 1.00]); and the interaction between 2w-diet and stress was statistically significant and large (F_(1,24)_ = 5.78, *p* = 0.024; η^2^ (partial) = 0.19, 95% CI [0.02, 1.00]).

#### 3.7.3. Accumbal D2R Expression of Ad Libitum and Fasted Animals from 2w-chow and 2w-pal-fed Groups

Figure 10C and Supplementary Figure S4 shows that D2R expression decreased in the 2w-chow-C-F group by 72.75% when compared to 2w-chow-S-Al, and in 2w-chow-S-F by 214.29% on average vs. all the other groups. The three-way ANOVA showed that the main effect of 2w-diet was statistically significant and large (F_(1,24)_ = 8.92, *p* = 0.006; η^2^ (partial) = 0.27, 95% CI [0.05, 1.00]); the main effect of fasting was statistically significant and large (F_(1,24)_ = 27.29, *p* < 0.001; η^2^ (partial) = 0.53, 95% CI [0.29, 1.00]); the interaction between 2w-diet and fasting was statistically significant and large (F_(1,24)_ = 13.59, *p* = 0.001; η^2^ (partial) = 0.36, 95% CI [0.12, 1.00]); and the interaction between fasting and stress was statistically significant and large (F_(1,24)_ = 4.88, *p* = 0.037; η^2^ (partial) = 0.17, 95% CI [6.20 × 10^−3^, 1.00]).

#### 3.7.4. Accumbal D2R Expression of 2w-chow and 2w-pal-fed Groups After Refeeding

Figure 10D and Supplementary Figure S4 depicts that after refeeding, the 2w-chow-S-Rc group showed increased expression of D2R in the NAc to 98.28% on average vs. all the other groups. The three-way ANOVA showed that the main effect of refeeding was statistically significant and large (F_(1,24)_ = 5.06, *p* = 0.034; η^2^ (partial) = 0.17, 95% CI [8.04 × 10^−3^, 1.00]); the main effect of stress was statistically significant and large (F_(1,24)_ = 25.05, *p* < 0.001; η^2^ (partial) = 0.51, 95% CI [0.26, 1.00]); the interaction between 2w-diet and refeeding was statistically significant and large (F_(1,24)_ = 5.47, *p* = 0.028; η^2^ (partial) = 0.19, 95% CI [0.01, 1.00]); the interaction between 2w-diet and stress was statistically significant and large (F_(1,24)_ = 23.32, *p* < 0.001; η^2^ (partial) = 0.49, 95% CI [0.24, 1.00]); and the interaction between refeeding and stress was statistically significant and large (F_(1,24)_ = 7.86, *p* = 0.010; η^2^ (partial) = 0.25, 95% CI [0.04, 1.00]).

## 4. Discussion

Chronic stress exposure is known to increase palatable food intake [15,21,28], and both stress and long-term high-energy food intake are considered etiological factors for the development of obesity and overweight [29,30]. A neural circuit recognized as having a role in the hyperphagia for palatable food is the one that includes cells of the PVN and PBN. Studies show that the acute intake of condensed milk is able to activate PVN^MC4R^ and cLPBN neurons [9,11]. This change induced by palatable food intake has been associated with reduced satiety in animals and may therefore contribute to the development of hyperphagia. However, the effects of chronic stress and long-term palatable food intake on the neural activation of this circuit have not been investigated. Likewise, it remains unclear how fasting, as a metabolic stimulus that directly affects PVN neurons, and subsequent refeeding influence the neural activity of specific cell populations within this circuit.

Therefore, in the present study, stressed and non-stressed animals were exposed for 2 w to either a standard chow or palatable food diet. We evaluated the neuronal activation of PVN cells expressing the MC4R using cFos-IR, as MC4R mediates the effects of the anorexigenic peptide α-MSH. We also assessed cFos activation in PVN neurons expressing TRH, which has also been implicated as an appetite-inhibiting factor. Additionally, we analyzed the changing patterns of cFos-neural activation in caudolateral PBN (cLPBN) cells expressing vGLUT2 or CRF, both of which have been implicated in anorexigenic signaling.

Our results showed that animals provided with chow plus chocolate milk consumed greater amounts of palatable food and exhibited an increase in b.w. gain than animals receiving only chow, consistent with previously published studies [22,28,31]. However, the higher energy intake from palatable food was not affected by chronic stress exposure, as previously described [22,32].

### 4.1. MC4R

The similar changes in the number of PVN^MC4R+cFos^ cells in the aPVN and mPVN of 2w-chow-fed rats following fasting and chronic stress exposure (relative to their ad libitum-fed, unstressed controls) suggested functional coordination between these subcompartments, despite their distinct projections (aPVN to the PBN, and mPVN to the DMV/NTS [33]). This coordinated activity may contribute to the regulation of neural circuits involved in feeding, satiety, energy expenditure, and body-weight regulation during negative energy balance (NEB).

The 2 w exposure to palatable food strikingly altered the number of activated cells in both PVN subnuclei, even in unstressed and ad libitum-fed conditions. These findings suggested that a 2 w exposure to a palatable food diet could impair the neural responsiveness of anorexigenic circuits involved in satiety regulation, potentially favoring animals’ hyperphagia for palatable food. Although further studies are needed to determine the underlying mechanisms, components of chocolate milk, such as palmitic acid, might contribute to this effect, since it decreases α-MSH synthesis and release [34,35] into the PVN. A similar adaptation in the responsiveness of these neural circuits might contribute to the development of positive energy balance by reducing cFos-defined recruitment of anorexigenic neuronal populations in response to feeding-related signals. Thus, food intake reduction and body weight loss might be hindered.

Refeeding the animals with either chow or palatable food provided an opportunity to assess the differential activation of PVN^MC4R+cFos^ cells in both subnuclei after 2 weeks of chow or pal-food intake. Our findings suggest that the activation of PVN^MC4R+cFos^ neurons during refeeding is not determined solely by the restoration of energy availability but is strongly influenced by prior dietary experience and by the interaction between stress and food type. Animals kept on chow for 2 weeks recovered PVN^MC4R+cFos^ activity after refeeding, regardless of whether they received chow or the palatable diet, indicating a robust reinstatement of melanocortin signaling following fasting. In contrast, 2 weeks of exposure to the palatable diet altered this response, as chow refeeding failed to restore PVN^MC4R+Fos^ cell activation to baseline levels. Interestingly, this blunted response was reversed when palatable food was available during refeeding under stress, suggesting that prior exposure to a palatable diet modifies the context-dependent responsiveness of PVN^MC4R^ neurons. Rather than reflecting a generalized impairment of melanocortin signaling, these observations support that PVN^MC4R^ neurons become selectively engaged according to the metabolic state, prior dietary experience, and the type of food available during refeeding.

This finding challenged the assumption that greater MC4R-cFos neuronal activation necessarily reduces food intake, as these animals consumed more palatable food. One possibility is that the increased activation represents a compensatory response especially when engaged during refeeding with palatable food.

### 4.2. pro-TRH

The differential activation of pro-TRH+cFos-expressing neurons in the anterior (aPVN) and medial (mPVN) paraventricular subnuclei suggests that these TRHergic populations contribute to distinct aspects of feeding regulation, as previously described [36]. The absence of a fasting-induced reduction in aPVN^pro-TRH^ activity in 2w-chow-fed animals supports the idea that the aPVN is relatively insensitive to NEB and may preferentially contribute to non-homeostatic components of feeding. In contrast, mPVN pro-TRH neurons were markedly sensitive to metabolic status, as both fasting and stress under NEB reduced the number of pro-TRH+cFos-expressing cells in chow-fed animals. This response is consistent with the well-established suppression of hypothalamic-pituitary-thyroid (HPT) axis activity during energy deficit. The heterogeneous expression of GR in pro-TRHergic neurons between the aPVN and mPVN may contribute to the attenuated or absent effects of stress observed in the aPVN [37].

Notably, chronic exposure to the palatable diet modified this physiological response. The reduced number of mPVN^pro-TRH+cFos^ neurons already observed in the 2w-pal-C-Al group suggests that prolonged consumption of energy-dense food resulted in a lower basal level of neuronal activation, thereby generating a floor effect that prevented an additional reduction normally induced by fasting or stress.

Nevertheless, stressed animals kept on the palatable diet exhibited greater body weight than their chow-fed counterparts, despite a similarly reduced mPVN^pro-TRH-cFos^ cells’ response. This dissociation suggests that reduced HPT axis activity alone is insufficient to account for the observed differences in body weight. Rather, in the context of chronic exposure to a highly energy-dense diet, reduced recruitment of mPVN pro-TRH neurons may be associated with metabolic adaptations that favor positive energy balance and increase susceptibility to obesity.

In 2w-chow-C-Al rats, the lower number of aPVN^pro-TRH-cFos^-positive cells compared with mPVN^pro-TRH-cFos^-positive cells was consistent with the functional compartmentalization of these subnuclei described above [35]. Whereas the number of cFos-expressing pro-TRHergic cells in the aPVN was not decreased by homeostatic challenges such as fasting and stress, their activation was selectively modulated by the type of food provided during refeeding.

Similarly, in 2w-chow-fed animals, the activation of mPVN pro-TRHergic neurons was modulated by the type of food provided during refeeding. The higher number of cFos-expressing pro-TRHergic neurons observed under these conditions may be related to the relatively low food intake observed in this group. In contrast, 2w-pal exposure blunted the responsiveness of pro-TRHergic neurons in both the aPVN and mPVN, as the number of cFos-expressing pro-TRHergic neurons remained reduced after refeedingwith either chow or palatable food. Together with the reduced recruitment of mPVN pro-TRHergic neurons following fasting and stress, this altered response pattern suggests that mPVN pro-TRHergic neurons may be more susceptible to the effects of prolonged palatable food exposure than their aPVN counterparts.

A notable finding was the differential cFos-defined recruitment of pro-TRH- and MC4R-expressing neurons in the aPVN of 2w-chow-C-Al animals following fasting and stress. Because neurons in the aPVN project to the PBN [13], these observations suggest that pro-TRH may not be the primary neuropeptide contributing to the recruitment of the PVN^MC4R^–PBN pathway during feeding-related challenges. However, because our study assessed cFos immunoreactivity without directly evaluating synaptic connectivity or neuronal projections, this interpretation remains speculative and will require direct circuit-level approaches to determine whether pro-TRH neurons participate in this pathway.

### 4.3. Integrative Role of the Parabrachial Nucleus in Processing Feeding and Stress-Related Signals

The role of the cLPBN in integrating homeostatic and hedonic signals to regulate food intake has been previously described [9]. Our findings are consistent with this proposed role, as the number of vGlut2- and CRF-cFos-positive cells differentially changed in the cLPBN of re-fed animals following a 48 h fasting period, depending on their previous dietary experience and the type of food provided during refeeding.

The almost negligible number of cLPBN^vGlut2+cFos^ and cLPBN^CRF+cFos^ neurons observed under control (unstressed)-ad libitum conditions in both the 2w-chow and pal-fed groups limited the possibility of detecting further reductions in response to stress or fasting. The absence of an increase in cLPBN^vGlut2+cFos^ and cLPBN^CRF+cFos^ cells in 2w-pal-C-Al animals compared with chow-fed controls suggested that chronic consumption of palatable food does not increase basal recruitment of these neuronal populations. This finding contrasts with the increased recruitment previously reported in the cLPBN following an acute consumption of highly-dense food [9], and supports a differential responsiveness depending on the chronicity of the stimulus. Whether this blunted basal recruitment contributes to sustained hyperphagia and obesity remains to be determined.

A modest but significant increase in the recruitment of cLPBN^vGlut2+cFos^ neurons was observed following fasting in animals previously exposed to the palatable diet for 2 weeks. A similar trend was detected in cLPBN^CRF+cFos^ neurons, although this change did not reach statistical significance. These observations suggest that prolonged exposure to palatable food may selectively alter the responsiveness of specific neuronal populations in the cLPBN to metabolic challenges, with fasting eliciting a response that was not observed following chronic stress alone. Thus, long-term consumption of a palatable diet may modify cLPBN neuronal responsiveness to NEB, consistent with a potential role for this parabrachial subregion in integrating metabolic signals associated with energy deficit.

Interestingly, although fasting is considered a physiological stressor that can elevate corticosterone serum levels, its effects on cLPBN^vGlut2+cFos^ and cLPBN^CRF+cFos^ neuronal recruitment differed from those produced by isolation stress. This divergence highlights that other molecules pertaining to the stress system, as well as the nature, intensity, and chronicity of the challenge, are relevant for delineating the response of those cLPBN cells [14,15,38].

Refeeding revealed that the responsiveness of cLPBN^vGlut2^ and cLPBN^CRF^-expressing neurons depends on both dietary history and stress context rather than on the refeeding stimulus alone. In chow-fed animals, refeeding with the palatable diet produced a modest increase in the recruitment of cLPBN^vGlut2-cFos^ neurons, whereas the response of cLPBN^CRF^ neurons was comparatively limited, suggesting that these neuronal populations are differentially engaged despite both expressing factors associated with anorexigenic signaling. Chronic stress further modified this pattern by preventing the increase in cLPBN ^vGlut2^ recruitment observed in chow-fed animals and by altering the recruitment of cLPBN^CRF^ neurons in animals previously exposed to the palatable diet. Together, these findings suggest that the cLPBN neuronal response to refeeding is modified according to the interaction between previous dietary experience, stress exposure, and the rewarding properties of the food, rather than encoding metabolic state in isolation [39].

The lack of stress-induced activation of cLPBN^CRF^ was striking, given that the CRFergic system is classically associated with responses to threatening stimuli. cLPBN^CRF+cFos^ neurons therefore appeared to have other functional roles than previously recognized.

Previous studies have shown that short-term consumption of palatable food activates cLPBN neurons in rodents [9]. Consistent with these findings, we observed an increment in the number of activated cLPBN^vGlut2+cFos^ cells when animals were re-fed with palatable food independently of their dietary history. This response was greater in animals exposed to palatable food for 2 weeks, indicating that palatable food consumption has a profound effect on these cells’ activation. In contrast, cLPBN^CRF+cFos^ neuronal activation increased only in 2w-pal-S-Rpal and not in 2w-chow-S-Rpal, which supports that even when CRF and vGlut2 signaling in the cLPBN are implicated in anorexigenic effects, their recruitment during refeeding is differentially influenced by prior dietary history.

The marked recruitment of cLPBN^vGlut2+Fos^ in 2w-pal-S-Rpal animals may represent a compensatory response to the high caloric intake associated with palatable food refeeding. However, this may also be reflecting a resistance to those peptides’ effects since animals still ate greater amounts of high energy dense food than those with a history of 2w-chow food intake. Regardless, this interpretation remains to be confirmed.

The greater cFos-defined recruitment of PBN vGlut2- and CRF-expressing neurons in chronically stressed animals, compared with unstressed animals re-fed the same palatable food, suggests that chronic stress alters the responsiveness of these parabrachial neuronal populations to hedonic feeding. Because fasting-induced stress alone produced a more modest response, these findings are consistent with the idea that chronic stress does not simply mimic the effects of acute NEB but enhances the recruitment of PBN neurons when palatable food is consumed during refeeding. This interaction between chronic stress and previous dietary experience may reflect a context-dependent modulation of parabrachial circuits involved in integrating metabolic and hedonic information.

Our results showed that the responsiveness pattern in cLPBN^vGlut2+cFos^ neurons of 2w-pal-fed groups paralleled that of PVN^MC4R^, whereas those of 2w-chow-fed animals displayed an activation pattern that resembled the one observed in aPVN^pro-TRH^ neurons in 2w-chow-S-Rc rats. It has to be elucidated whether those neurons independently project to the PBN as previously recognized for PVN^MC4R^ neurons and whether these inputs form functional synaptic connections with PBN^vGlut2^ cells, thus tuning their response to stimuli of a diverse nature and regulating feeding behavior differentially.

### 4.4. Changes in VTA Tyrosine Hydroxylase and in NAc D2R Expression

cLPBN neurons are known to project to the VTA and may modulate dopaminergic signaling and feeding behavior [11]. The NAc, in turn, receives VTA DAergic projections, and it is also involved in eating regulation; thus, we evaluated VTA TH and NAc D2R expression in all groups.

TH and D2R expression also differed according to the animals’ dietary experience. In 2w-chow-diet groups, the combination of fasting, stress, and chow-food refeeding was associated with decreased TH and D2R expression, whereas this response was blunted in animals previously exposed to the palatable diet. The specific increase in D2R expression by S-Rc was also prevented by the intake of 2w-pal-diet, suggesting that chronic palatable food exposure history impacts the adaptive responsiveness of the mesolimbic reward system to food’s hedonic properties.

These results are in line with the proposed activation of the dopaminergic system induced by stress as previously reported in the same paradigm [32]. As preference for pal-food over that of chow increased in 2w-pal-fed animals, an altered plasticity might impair the ability to appropriately adjust reward-related signaling to food intake, potentially contributing to reduced control over pal-food consumption.

### 4.5. Limitations

This study has several limitations. cFos immunoreactivity and endpoint protein expression are indirect and temporally limited measures; they indicate recent neuronal activity but not neuronal output, neurotransmitter release, or functional connectivity. The functional connectivity along the PVN–PBN–VTA–NAc axis was inferred rather than directly demonstrated, as no viral tracing, optogenetics, chemogenetics, or electrophysiology was performed. Only male rats were used. Given well-established sex differences in feeding behavior, obesity susceptibility, stress responsiveness, hypothalamic regulation, and dopaminergic signaling [40,41,42,43], our findings may not generalize to females. Thus, future studies including both sexes are needed to determine whether these effects are sex specific. As prolonged fasting can also induce broader metabolic and neuroendocrine adaptations that may contribute to the observed effects, our findings should be interpreted within the context of prolonged fasting and refeeding, rather than as a direct representation of physiological feeding regulation.

## 5. Conclusions

Our findings provide evidence that prolonged exposure to an energy-dense palatable diet modifies the recruitment of neuronal populations within the PVN and PBN that express receptors or neuropeptides implicated in anorexigenic regulation.

These alterations were observed even in animals that had not yet developed marked increases in body weight, suggesting that neural adaptations may precede overt obesity. Chronic isolation stress further modified the recruitment of these neuronal populations, indicating an interaction between dietary history and stress in changing central responses to metabolic and hedonic stimuli.

In particular, long-term exposure to palatable food altered the recruitment of PVN^MC4R^, PVN^pro-TRH^, PBN^vGlut2^, and PBN^CRF^ neurons during fasting, stress, and refeeding. The selective recruitment of PBN vGlut2- and CRF-expressing neurons according to dietary history is consistent with differential engagement of parabrachial pathways implicated in satiety, aversive processing, and mesolimbic reward circuits. Together, these findings are consistent with circuit-level plasticity, through which chronic palatable food exposure, further influenced by chronic stress, may alter the integration of homeostatic, hedonic, and reward-related signals that regulate feeding behavior.

## Figures and Tables

**Figure 1:**
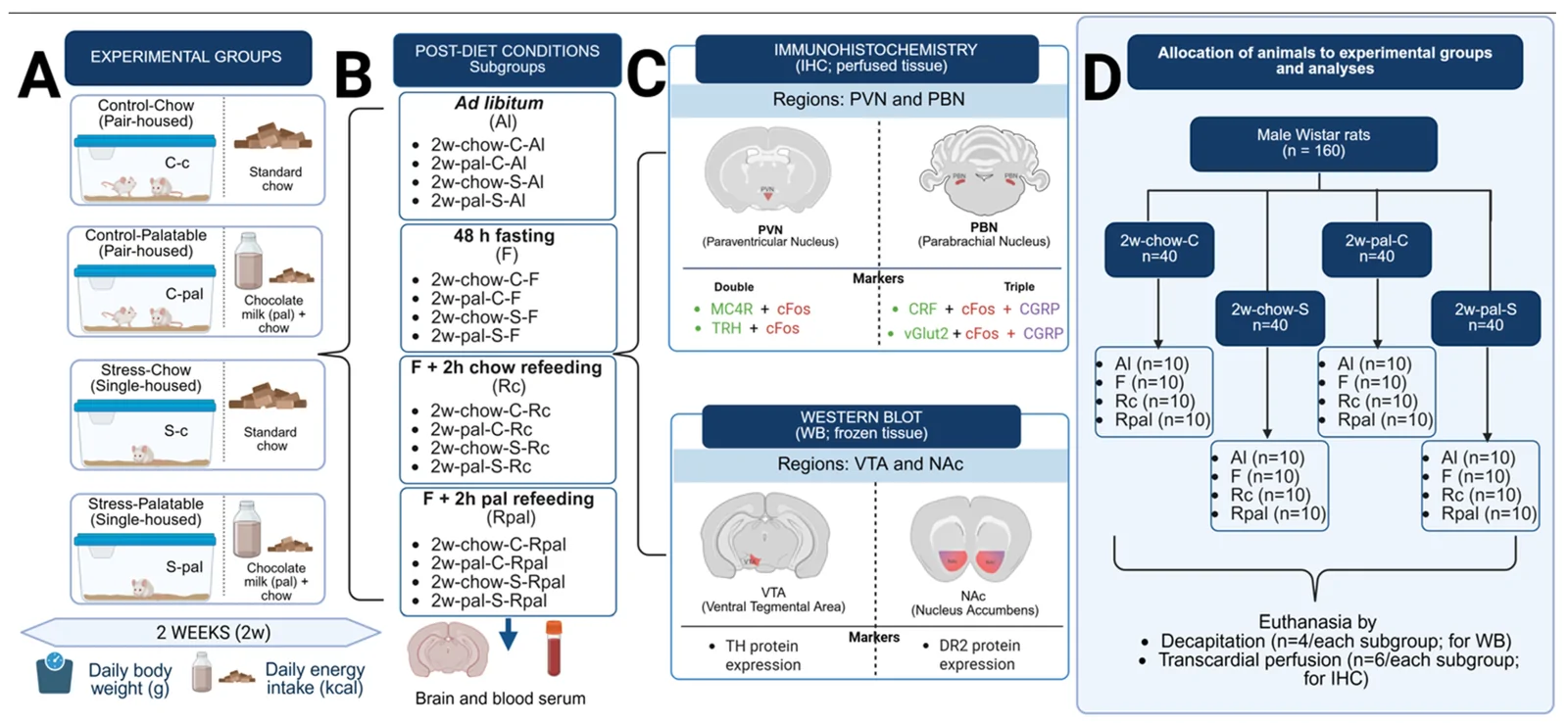
Experimental design. (**A**) Adult male rats were housed either in pairs (control, C) or individually (stressed, S) and fed for 2 weeks (2w) either standard chow (C-c, S-c) or chow plus chocolate milk (C-pal, S-pal). (**B**) After 2 weeks, rats were subdivided into four refeeding conditions: ad libitum (Al), 48-h fasting (F), or F + re-fed for 2 h with chow (Rc), or F + re-fed for 2 h with chocolate milk (Rpal), after which they were euthanized. (**C**) Perfused brains were processed for double immunohistochemistry (IHC: cFos + MC4R or pro-TRH) in the paraventricular nucleus (PVN), and triple immunofluorescence (cFos + CGRP + vGlut2 or CRF) in the parabrachial nucleus (PBN). Frozen brains were dissected to obtain ventral tegmental area (VTA) and nucleus accumbens (NAc) for Western blot analysis of TH and D2R, respectively. (**D**) Allocation and number of animals to each experimental group. Created with BioRender.

**Figure 2:**
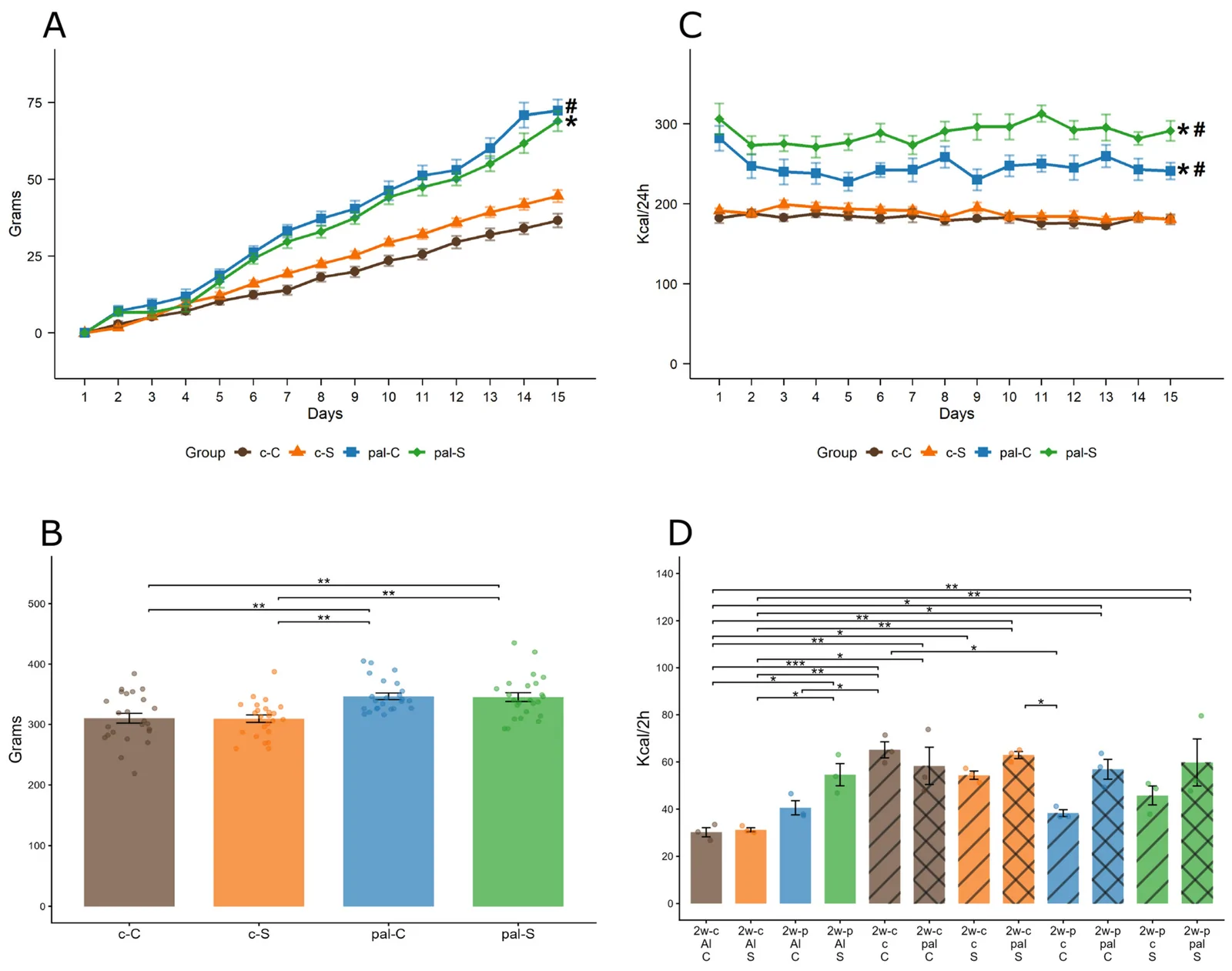
Effect of palatable food intake and chronic stress exposure on body weight and energy intake. Panel (**A**) depicts the daily weight gain of rats maintained in control or stress conditions and fed with either chow (ochre shades) or chocolate milk plus standard chow (blue/green shades). Daily weight gain was calculated as the difference between the weight registered on one day minus that of the day before; data are the mean ± SEM of the daily weight gain in grams of each group: c-C (fed with chow for 2 weeks and pair-housed), c-S (fed with chow for 2 weeks and single-housed), pal-C (fed with chocolate milk plus chow for 2 weeks and pair-housed), pal-S (fed with chocolate milk plus chow for 2 weeks and single-housed); n = 24/group. * *p* < 0.001 vs. c-C and # *p* < 0.001 vs. c-S. Panel (**B**) shows the final body weight at the end of the 2 weeks of the four described groups; data are expressed as mean ± SEM of the b.w. in grams, n = 24/group. Panel (**C**) shows daily food intake over the 2 weeks of the experiment of the four groups described above. As control animals were housed in pairs, the food intake of each cage was considered as one data point; in contrast, daily food consumption of 2 single-housed (stressed) rats was randomly summed to be compared with controls (n = 12/group). Data are the mean ± SEM of the daily ingested kcal/cage/group for 2 weeks. * *p* < 0.001 vs. c-C and # *p* < 0.001 vs. c-S. Panel (**D**) shows 2 h caloric intake after the beginning of the activity phase. Rats were fed ad libitum (Al) with standard chow (2w-c, in ochre shades) or chocolate milk as palatable food plus chow (2w-p, in blue/green shades) for 2 weeks. Rats were maintained ad libitum (Al, solid bars) or were fasted for 48 h and then re-fed with either chow (c) or chow plus palatable food (pal) (patterned bars). Rats from all conditions were either pair-housed (C) or single-housed (S). Because control animals were pair-housed, food intake was measured per cage (2 rats) and considered as a single data point. Accordingly, food intake from two randomly selected single-housed (stressed) rats was summed to generate a comparable data point. Data are presented as the mean ± SEM of the ingested kcal/cage/group during the 2 h of the refeeding period (n = 3/group). * *p* < 0.05, ** *p* < 0.01, *** *p* < 0.001 vs. depicted groups, obtained by Tukey post-hoc tests.

**Figure 3:**
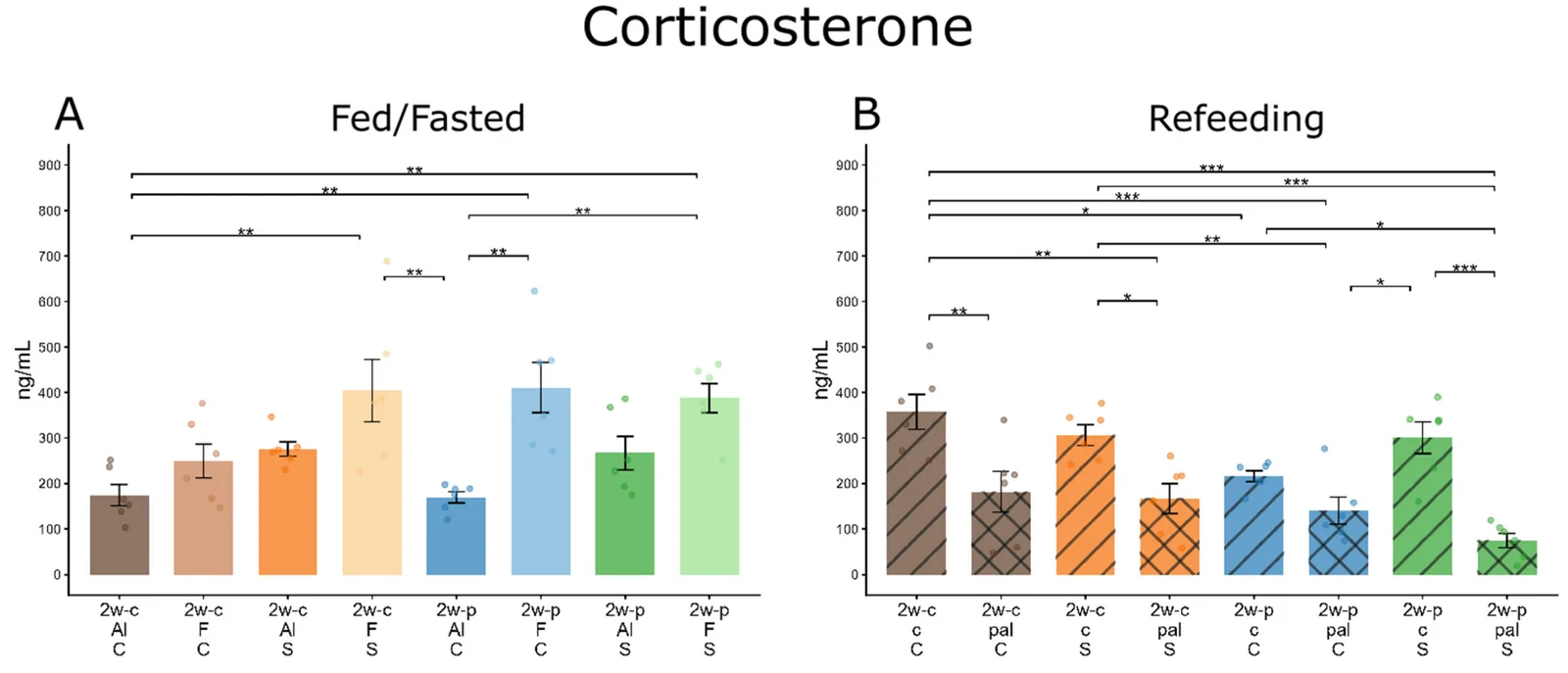
Effect of pal-food intake, fasting, and refeeding on corticosterone serum levels. Panel (**A**) depicts the corticosterone serum levels of rats that were fed ad libitum (Al) with standard chow (2w-c, in ochre shades) or chocolate milk as palatable food plus chow (2w-p, in blue/green shades) for 2 weeks, and those that were fasted for 48 h (F). Rats from the Al and F conditions were either pair-housed (C) or single-housed (S) (solid bars). Panel (**B**) includes animals from the conditions described for panel (**A**) that were re-fed after F with either chow (c) or palatable food plus chow (pal) (patterned bars). n = 6/group. Data are the mean ± SEM of serum corticosterone levels expressed in ng/mL. * *p* < 0.05, ** *p* < 0.01, *** *p* < 0.001 vs. depicted groups, obtained by Tukey post-hoc tests.

**Figure 4:**
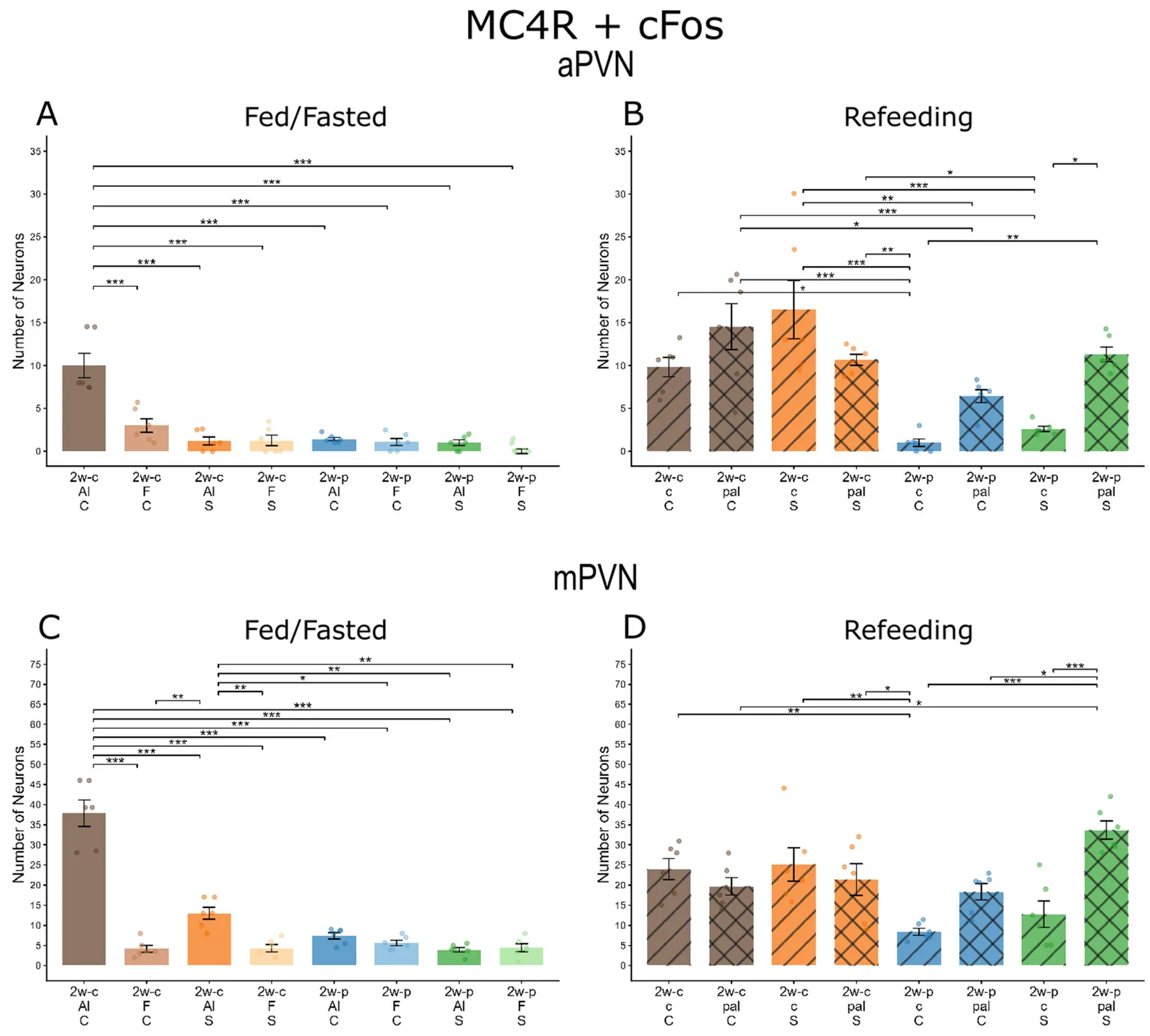
Effect of pal-food intake and chronic stress exposure on the cFos-defined activation of MC4R neurons in the PVN. Panels (**A**,**B**) show the number of activated cells in the aPVN^MC4R+cFos^. Panel (**A**) depicts rats that were fed ad libitum (Al) with standard chow (2w-c, in ochre shades) or chocolate milk as palatable food plus chow (2w-pal, in blue/green shades) for 2 weeks, and those that were fasted for 48 h (F). Rats from the Al and F conditions were either pair-housed (C) or single-housed (S) (solid bars). Panel (**B**) includes animals from the conditions described for panel (**A**) that were re-fed after fasting with either chow (c) or palatable food plus chow (pal) (patterned bars). Panels (**C**,**D**) show the number of activated cells in the mPVN^MC4R+cFos^. Panel (**C**) depicts rats that were fed ad libitum (Al) with standard chow (2w-c) or chocolate milk as palatable food plus chow (2w-p) for 2 weeks, and those that were fasted for 48 h (F). Rats from the Al and F conditions were either pair-housed (C) or single-housed (S) (solid bars). Panel (**D**) includes animals from the conditions described for panel (**A**) that were re-fed after F with either chow (c) or palatable food plus chow (pal) (patterned bars). Data are presented as the mean ± SEM; n = 6/group. * *p* < 0.05, ** *p* < 0.01, *** *p* < 0.001, obtained by Tukey post-hoc tests.

**Figure 5:**
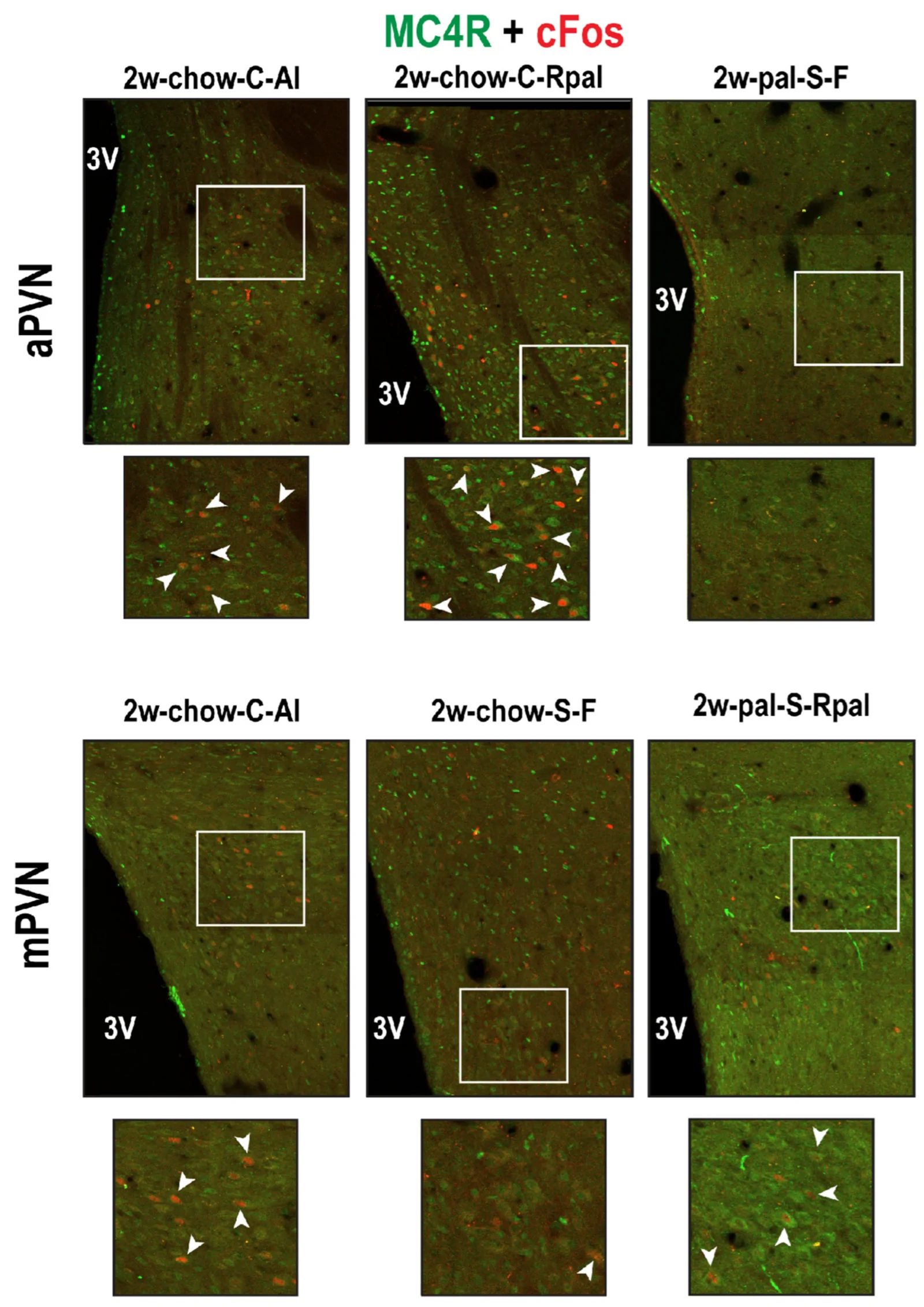
Representative photomicrographs of MC4R-activated neurons in the aPVN and mPVN of rats. Images are 20× magnifications of the anterior and medial subdivisions of the PVN of representative experimental groups, indicating MC4R (green channel) and cFos (red channel) positive cells. Arrowheads depict double-labeled cells. 3V: third ventricle.

**Figure 6:**
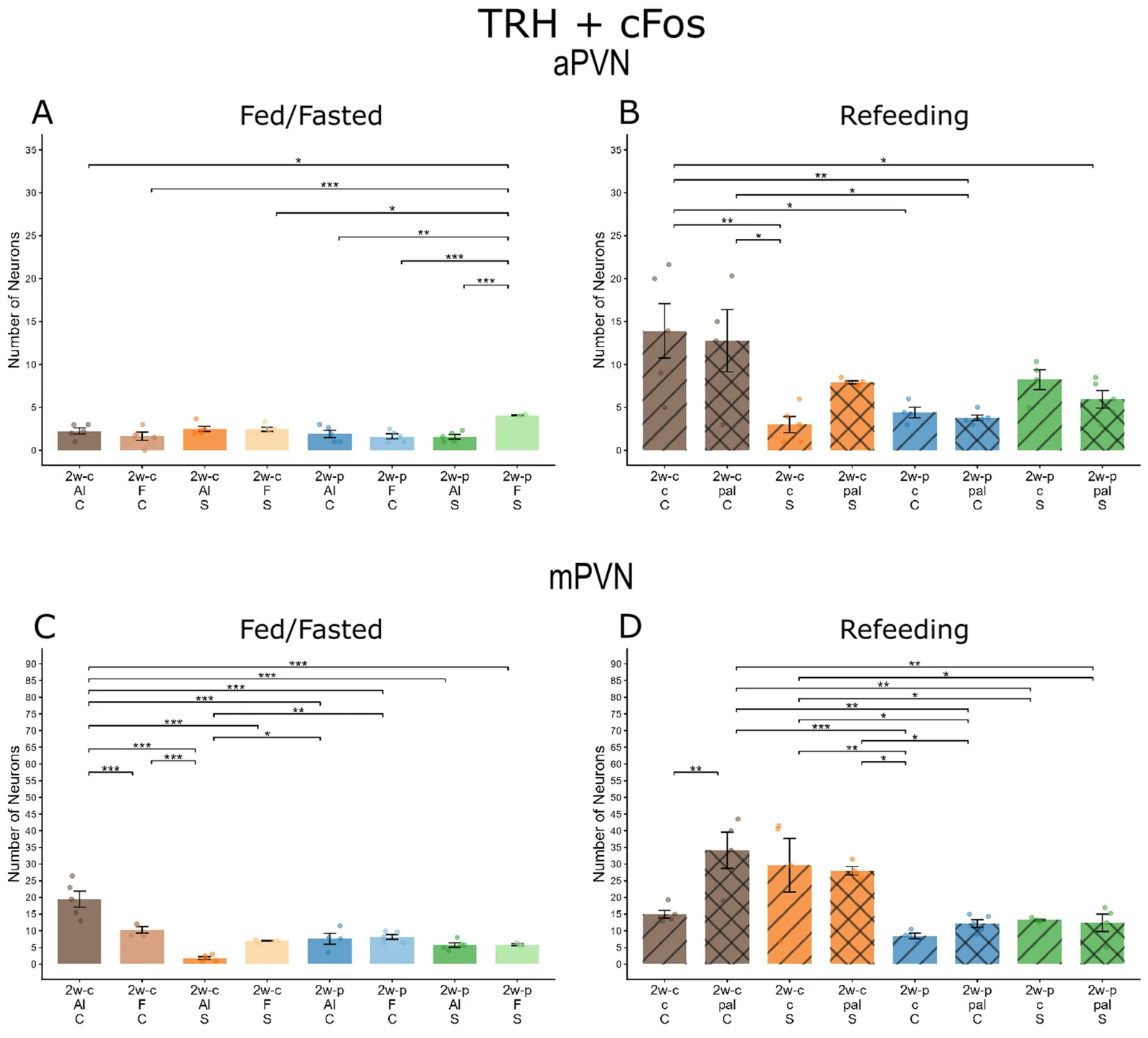
Effect of pal-food intake and chronic stress exposure on the cFos-defined activation of TRHergic neurons in the PVN. Panels A and B show the number of activated cells in the aPVN^TRH+cFos^. Panel (**A**) depicts rats that were fed ad libitum (Al) with standard chow (2w-c, in ochre shades) or chocolate milk as palatable food plus chow (2w-p, in blue/green shades) for 2 weeks, and those that were fasted for 48 h (F). Rats from the Al and F conditions were either pair-housed (C) or single-housed (S) (solid bars). Panel (**B**) includes animals from the conditions described for panel (**A**) that were re-fed after fasting, with either chow (c) or palatable food plus chow (pal) (patterned bars). Panels C and D show the number of activated cells in the mPVN^TRH+cFos^. Panel (**C**) depicts rats that were fed ad libitum (Al) with standard chow (2w-c) or chocolate milk as palatable food plus chow (2w-p) for 2 weeks, and those that were fasted for 48 h (F). Rats from the Al and F conditions were either pair-housed (C) or single-housed (S) (solid bars). Panel (**D**) includes animals from the conditions described for panel (**A**) that were re-fed after fasting with either chow (c) or palatable food plus chow (pal) (patterned bars). Data are presented as the mean ± SEM; n = 6/group. * *p* < 0.05, ** *p* < 0.01, *** *p* < 0.001, obtained by Tukey post-hoc tests.

**Figure 7:**
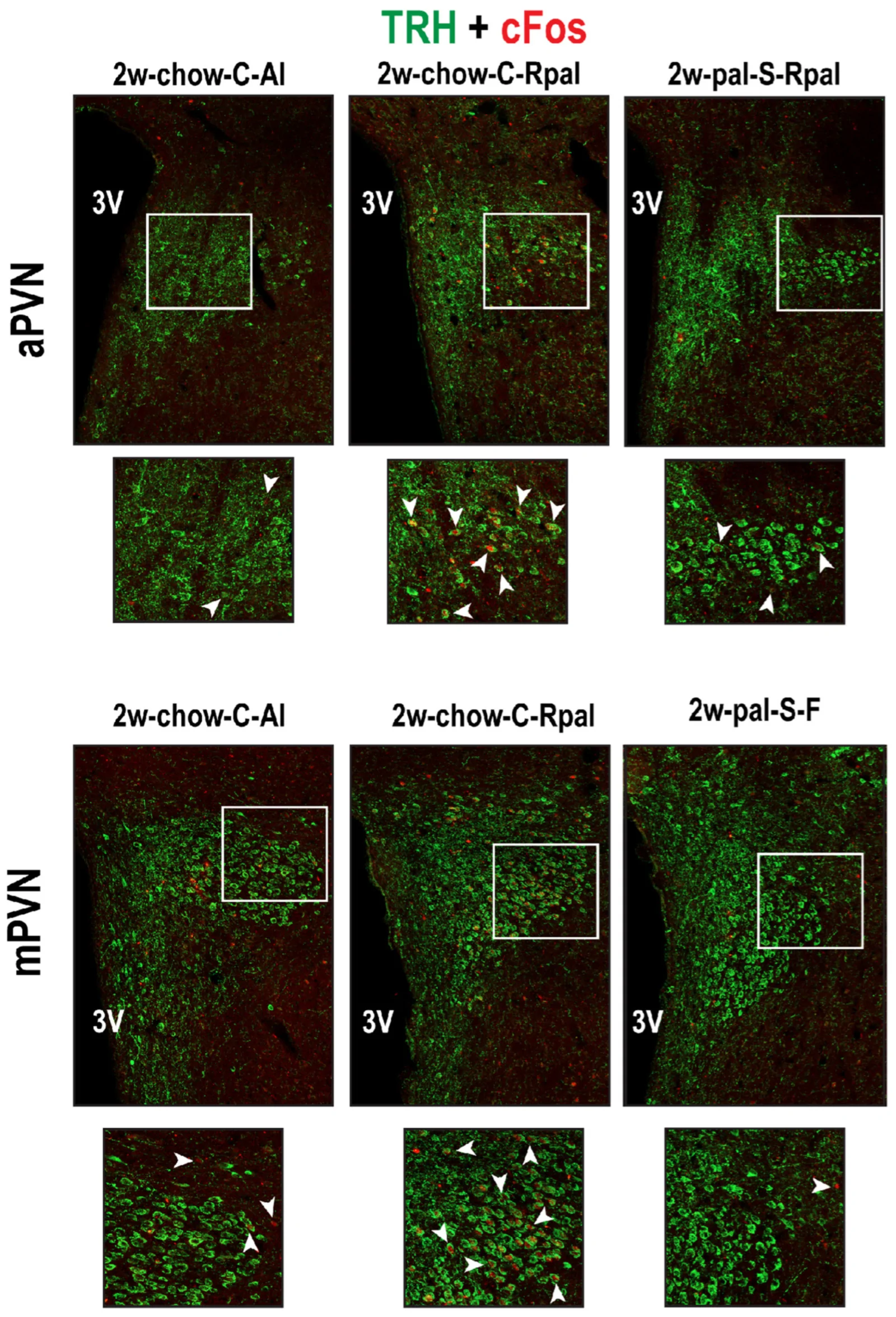
Representative photomicrographs of pro-TRH-activated neurons in the aPVN and mPVN of rats. Images are 20× magnifications of the anterior and medial subdivisions of the PVN of representative experimental groups, indicating pro-TRH (green channel) and cFos (red channel) positive cells. Arrowheads depict double-labeled cells. 3V: third ventricle.

**Figure 8:**
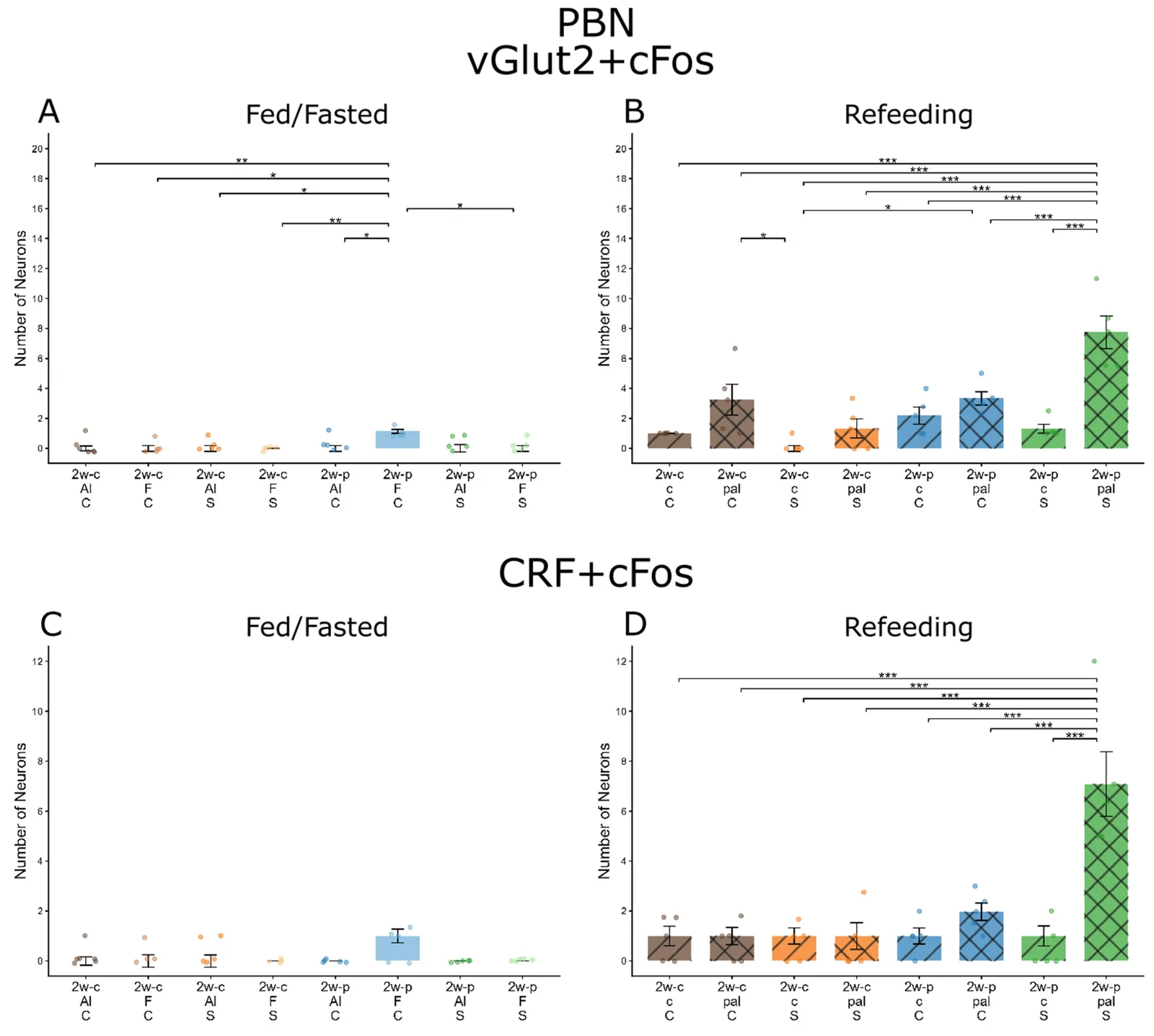
Effect of pal-food intake and chronic stress exposure on the activation of CRF and vGlut2 neurons in the PBN. Panels (**A**,**B**) show activated vGlut neurons in the PBN^vGlut+cFos^. Panel (**A**) depicts rats that were fed ad libitum (Al) with standard chow (2w-c, in ochre shades) or chocolate milk as palatable food plus chow (2w-p, in blue/green shades) for 2 weeks, and those that were fasted for 48 h (F). Rats from the Al and F conditions were either pair-housed (C) or single-housed (S) (solid bars). Panel (**B**) includes animals from the conditions described for panel (**A**) that were re-fed after fasting with either chow (c) or palatable food plus chow (pal) (patterned bars). Panels (**C**,**D**) show activated CRF neurons in the PBN^CRF+cFos^. Panel (**C**) depicts rats that were fed ad libitum (Al) with standard chow (2w-c) or chocolate milk as palatable food plus chow (2w-p) for 2 weeks, and those that were fasted for 48 h (F). Rats from the Al and F conditions were either pair-housed (C) or single-housed (S) (solid bars). Panel (**D**) includes animals from the conditions described for panel (**A**) that were re-fed after F with either chow (c) or palatable food plus chow (pal) (patterned bars). Data are presented as the mean ± SEM; n = 4–5/group, * when *p* < 0.05, ** *p* < 0.01, *** *p* < 0.001, obtained by the Tukey post-hoc test.

**Figure 9:**
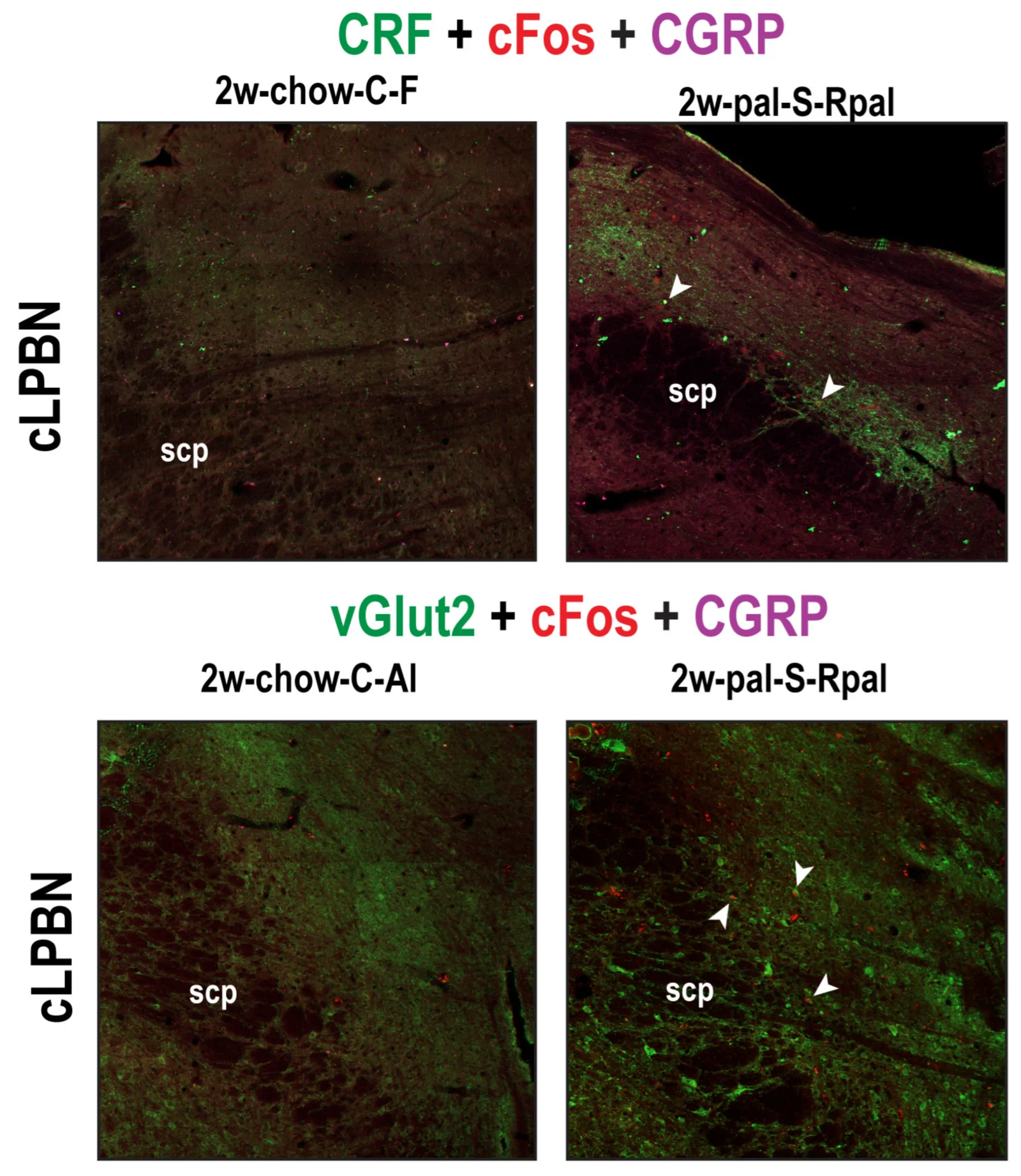
Representative photomicrographs of CRF- and vGlut2-activated neurons in the cLPBN of rats. Images are 20× magnifications of the caudolateral subdivision of the PBN of representative experimental groups, indicating CRF- or vGlut2 (green channel), cFos (red channel) positive cells, and negative for CGRP (magenta channel). Arrowheads depict double-labeled cells. Scp: superior cerebellar peduncle.

**Figure 10:**
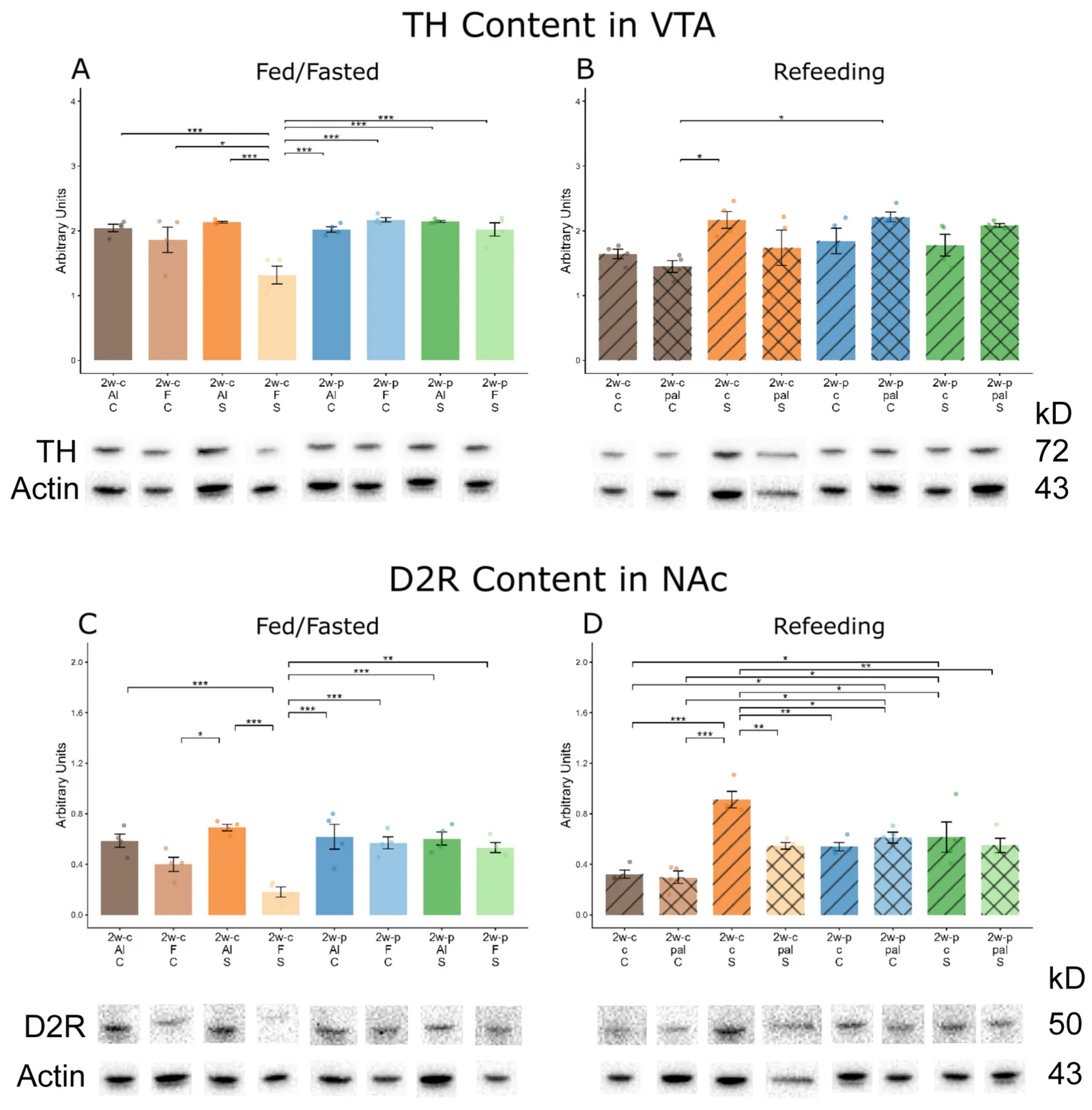
Effect of pal-food intake and chronic stress exposure on tyrosine hydroxylase and type-2 dopamine receptor in the VTA and NAc, respectively. Panel (**A**) depicts changes in the expression of tyrosine hydroxylase (TH) in the VTA of rats that were fed ad libitum (Al) with standard chow (2w-c, ochre shades) or chocolate milk as palatable food plus chow (2w-p, blue/green shades) for 2 weeks, and those that were fasted for 48 h (F). Rats from the Al and F conditions were either pair-housed (C) or single-housed (S) (solid bars). Panel (**B**) includes TH expression of animals from the conditions described for panel (**A**) that were re-fed after F with either chow (c) or palatable food plus chow (pal) (patterned bars). Panel (**C**) depicts changes in the expression of type-2 dopamine receptor (D2R) in the NAc of rats that were fed ad libitum (Al) with standard chow (2w-c) or chocolate milk as palatable food plus chow (2w-p) for 2 weeks, and those that were fasted for 48 h (F). Rats from the Al and F conditions were either pair-housed (C) or single-housed (S) (solid bars). Panel (**D**) includes D2R expression of animals from the conditions described for panel (**A**) that were re-fed after fasting, with either chow (c) or palatable food plus chow (pal) (patterned bars). Data are presented as the mean ± SEM of the ratio of the TH/actin or D2R/actin density obtained by Western blot, in arbitrary units, n = 4/group. * when *p* < 0.05, ** *p* < 0.01, *** *p* < 0.001, obtained by the Tukey post-hoc test.

## Data Availability

The raw data supporting the conclusions of this article will be made available by the authors on request.

## Supplementary Materials

The following supporting information can be downloaded at: https://www.mdpi.com/article/10.3390/brainsci16101111/s1. Supplementary Figure S1: Effect of pal-food intake and chronic stress exposure on the total number of MC4R-positive cells in the PVN; Supplementary Figure S2: Effect of pal-food intake and chronic stress exposure on the total number of TRH-positive cells in the PVN; Supplementary Figure S3: Effect of pal-food intake and chronic stress exposure on the total number of cFos-positive cells in the PBN; Supplementary Figure S4: Uncropped Western blot membranes showing target protein expression in the nucleus accumbens.

## Abbreviations

The following abbreviations are used in this manuscript:

PVN	Hypothalamic paraventricular nucleus
cLPBN	Caudolateral parabrachial nucleus
VTA	Ventral tegmental area
MC4R	Type-4 α-melanocortin-stimulating hormone receptor
D2R	dopamine receptor type 2
α-MSH	α-melanocortin-stimulating hormone
ARC	Arcuate nucleus
pro-TRH	pro-Thyrotropin releasing hormone
CRF	Corticotropin-releasing factor
CGRP	Calcitonin gene-related peptide
