# Supplementary material for: Homeostatic and Hedonic Signals Differentially Regulate cFos Activation in PVN MC4R, PVN pro-TRH, PBN vGlut2, and PBN CRF Neuronal Populations
Source: Brain Sci. 2026 Oct 15;16(10):1111. doi: 10.3390/brainsci16101111 (PMC13702733; doi:10.3390/brainsci16101111)
Supplement: Supplementary file 1 [file brainsci-16-01111-s001.zip › brainsci-4556752-supplementary.pdf]

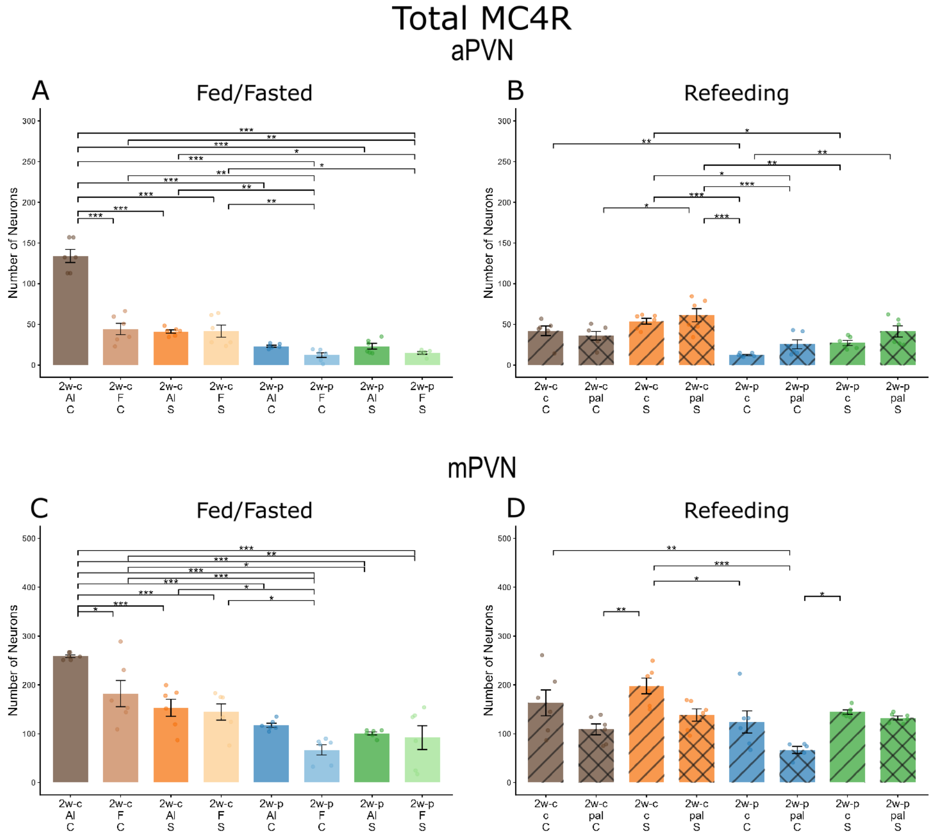

Supplementary Figure S1. Effect of pal-food intake and chronic stress exposure on the total number of MC4R-positive cells in the PVN. Panels A and B show the total number of aPVN^{MC4R}-positive cells. Panel A depicts rats that were fed ad libitum (Al) with standard chow (2w-c, in ochre tones) or chocolate milk as palatable food plus chow (2w-pal, in blue-green tones) for 2 weeks, and those that were fasted for 48 h (F). Rats from the Al and F conditions were either pair-housed (C) or single-housed (S) (solid bars). Panel B includes animals from the conditions described for panel A that were re-fed after F with either chow (c) or palatable food plus chow (pal) (patterned bars). Panels C and D show the total number of MC4R-positive cells in the mPVN. Panel C depicts rats that were fed *ad libitum* (Al) with standard chow (2w-c) or chocolate milk as palatable food plus chow (2w-p) for 2 weeks, and those that were fasted for 48 h (F). Rats from the Al and F conditions were either pair-housed (C) or single-housed (S) (solid bars). Panel D includes animals from the conditions described for panel A that were re-fed after F with either chow (c) or palatable food plus chow (pal) (patterned bars). Data are presented as the mean ± SEM; n = 6/group. *p < 0.05, **p < 0.01, ***p < 0.001, obtained by Tukey post-hoc tests.

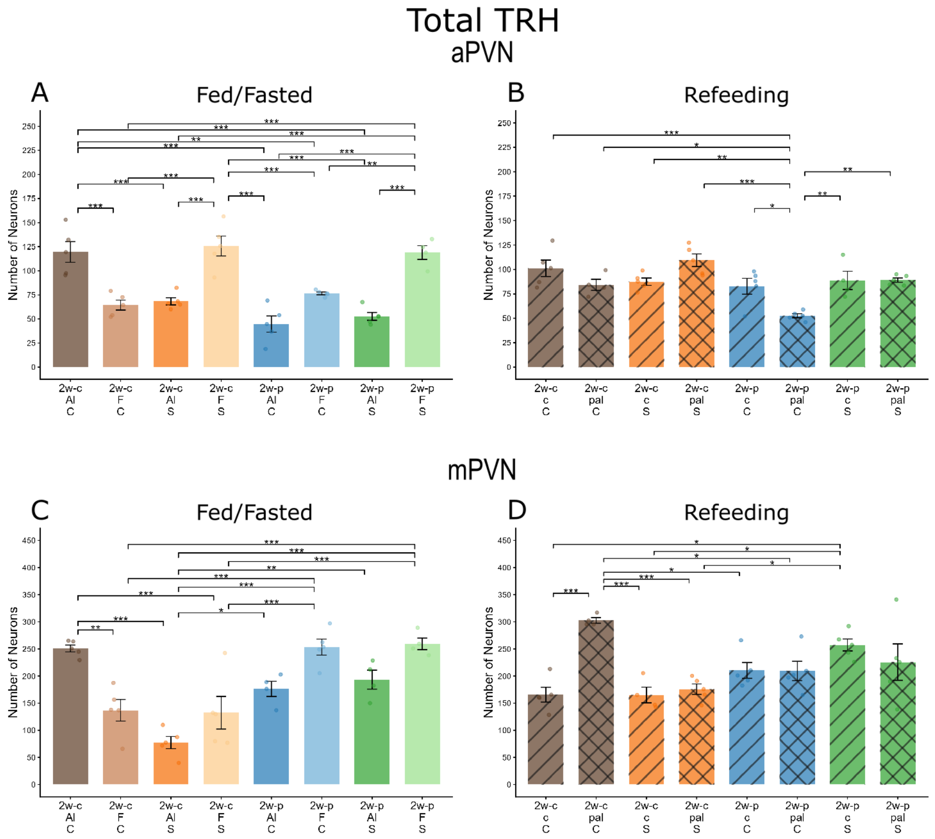

Supplementary Figure S2. Effect of pal-food intake and chronic stress exposure on the total number of MC4R-positive cells in the PVN. Panels A and B show the total number of aPVN^{TRH}-positive cells. Panel A depicts rats that were fed ad libitum (Al) with standard chow (2w-c, in ochre tones) or chocolate milk as palatable food plus chow (2w-pal, in blue-green tones) for 2 weeks, and those that were fasted for 48 h (F). Rats from the Al and F conditions were either pair-housed (C) or single-housed (S) (solid bars). Panel B includes animals from the conditions described for panel A that were re-fed after F with either chow (c) or palatable food plus chow (pal) (patterned bars). Panels C and D show the total number of TRH-positive cells in the mPVN. Panel C depicts rats that were fed *ad libitum* (Al) with standard chow (2w-c) or chocolate milk as palatable food plus chow (2w-p) for 2 weeks, and those that were fasted for 48 h (F). Rats from the Al and F conditions were either pair-housed (C) or single-housed (S) (solid bars). Panel D includes animals from the conditions described for panel A that were re-fed after F with either chow (c) or palatable food plus chow (pal) (patterned bars). Data are presented as the mean ± SEM; n = 6/group. *$p < 0.05$, **$p < 0.01$, ***$p < 0.001$, obtained by Tukey post-hoc tests.

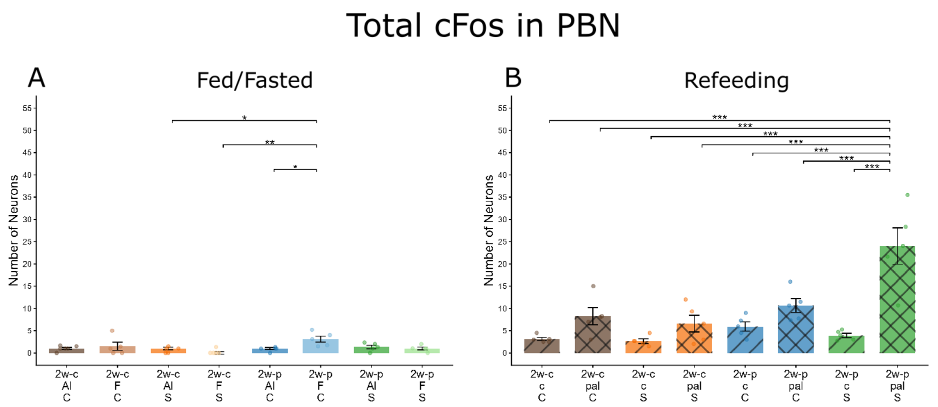

Supplementary Figure S3. Effect of pal-food intake and chronic stress exposure on the total number of cFos-positive cells in the PBN. Panels A and B show the total number of PBN^{cFos}-positive cells. Panel A depicts rats that were fed ad libitum (Al) with standard chow (2w-c, in ochre tones) or chocolate milk as palatable food plus chow (2w-pal, in blue-green tones) for 2 weeks, and those that were fasted for 48 h (F). Rats from the Al and F conditions were either pair-housed (C) or single-housed (S) (solid bars). Panel B includes animals from the conditions described for panel A that were re-fed after F with either chow (c) or palatable food plus chow (pal) (patterned bars). Data are presented as the mean ± SEM; n = 6/group. *p < 0.05, **p < 0.01, ***p < 0.001, obtained by Tukey post-hoc tests.

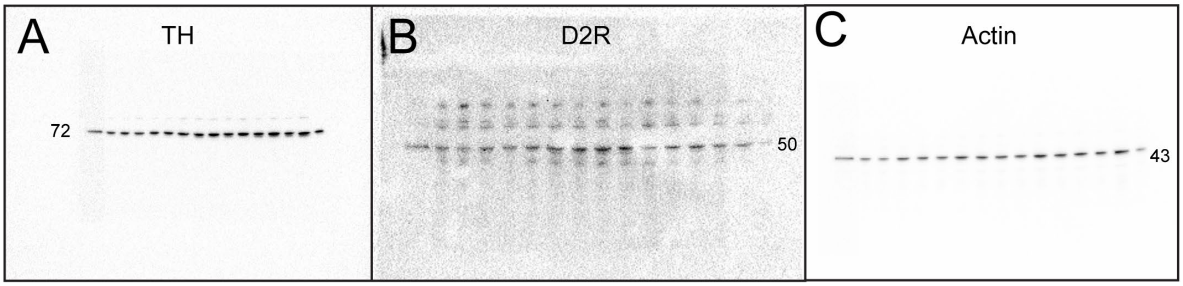

Supplementary Figure S4. Uncropped Western blot membranes showing target protein expression in the nucleus accumbens. Representative full-length immunoblots indicating the expression of tyrosine hydroxylase (TH, 72 kDa) in panel A, dopamine D2 receptor (D2R, 50 kDa) in panel B, and β-actin (loading control, 43 kDa) in panel C, in tissue lysates from the nucleus accumbens.
